# Hypertrophic cardiomyopathy mutations increase myofilament Ca^2+^ buffering, alter intracellular Ca^2+^ handling, and stimulate Ca^2+^-dependent signaling

**DOI:** 10.1074/jbc.RA118.002081

**Published:** 2018-05-14

**Authors:** Paul Robinson, Xing Liu, Alexander Sparrow, Suketu Patel, Yin-Hua Zhang, Barbara Casadei, Hugh Watkins, Charles Redwood

**Affiliations:** From the Cardiovascular Medicine Division, Radcliffe Department of Medicine, University of Oxford, Oxford OX3 9DU, United Kingdom

**Keywords:** cardiomyopathy, troponin, tropomyosin, Ca2+/calmodulin-dependent protein kinase II (CaMKII), extracellular-signal-regulated kinase (ERK), NFAT transcription factor, SERCA

## Abstract

Mutations in thin filament regulatory proteins that cause hypertrophic cardiomyopathy (HCM) increase myofilament Ca^2+^ sensitivity. Mouse models exhibit increased Ca^2+^ buffering and arrhythmias, and we hypothesized that these changes are primary effects of the mutations (independent of compensatory changes) and that increased Ca^2+^ buffering and altered Ca^2+^ handling contribute to HCM pathogenesis via activation of Ca^2+^-dependent signaling. Here, we determined the primary effects of HCM mutations on intracellular Ca^2+^ handling and Ca^2+^-dependent signaling in a model system possessing Ca^2+^-handling mechanisms and contractile protein isoforms closely mirroring the human environment in the absence of potentially confounding remodeling. Using adenovirus, we expressed HCM-causing variants of human troponin-T, troponin-I, and α-tropomyosin (R92Q, R145G, and D175N, respectively) in isolated guinea pig left ventricular cardiomyocytes. After 48 h, each variant had localized to the I-band and comprised ∼50% of the total protein. HCM mutations significantly lowered the *K_d_* of Ca^2+^ binding, resulting in higher Ca^2+^ buffering of mutant cardiomyocytes. We observed increased diastolic [Ca^2+^] and slowed Ca^2+^ reuptake, coupled with a significant decrease in basal sarcomere length and slowed relaxation. HCM mutant cells had higher sodium/calcium exchanger activity, sarcoplasmic reticulum Ca^2+^ load, and sarcoplasmic/endoplasmic reticulum calcium ATPase 2 (SERCA2) activity driven by Ca^2+^/calmodulin-dependent protein kinase II (CaMKII) phosphorylation of phospholamban. The ryanodine receptor (RyR) leak/load relationship was also increased, driven by CaMKII-mediated RyR phosphorylation. Altered Ca^2+^ homeostasis also increased signaling via both calcineurin/NFAT and extracellular signal–regulated kinase pathways. Altered myofilament Ca^2+^ buffering is the primary initiator of signaling cascades, indicating that directly targeting myofilament Ca^2+^ sensitivity provides an attractive therapeutic approach in HCM.

## Introduction

Hypertrophic cardiomyopathy (HCM)^4^ is an autosomal dominant disorder, usually caused by a single heterozygous genetic variant shared by all affected family members. It is the most common inherited cardiac disorder with a prevalence of 1 in 500 (1) and the leading cause of sudden death in young adults and athletes in particular (2). Mutations underlying the disease are principally found in genes that encode components of the contractile apparatus (3). The most commonly affected genes encode the thick filament proteins myosin-binding protein C (MyBPC) and β-myosin heavy chain (MyHC), whereas other HCM genes encode the thin filament regulatory proteins cardiac troponin T (cTnT), cardiac troponin I (cTnI), and α-tropomyosin (α-TM). We and others have established that HCM mutations in thin filament regulatory proteins increase myofilament Ca^2+^ sensitivity of actomyosin ATPase activity, *in vitro* motility and force of skinned muscle fibers (4–7). Furthermore, we have shown that this is due to an increase in actual Ca^2+^ affinity of the low-affinity regulatory Ca^2+^-binding site of cTnC (8, 9). Other HCM mutations (*e.g.* in *MYH7* (10) and *MYBPC3* (11)) are also predicted to increase troponin C Ca^2+^ binding caused by the effects of additional cross-bridge formation on cooperative thin filament activation. Thus, heightened myofilament Ca^2+^ sensitivity is likely to be a consistent feature of HCM mutations. Troponin C is the principal dynamic buffer of cytoplasmic Ca^2+^ and has been estimated to bind approximately half of the Ca^2+^ ions released by the sarcoplasmic reticulum (SR) during systole (12). We predict that the increased myofilament Ca^2+^ affinity will directly alter intracellular Ca^2+^ homeostasis in patients with HCM via increasing myofilament Ca^2+^ buffering. Increased buffering would cause deleterious changes to intracellular Ca^2+^ cycling, which may trigger Ca^2+^-dependent hypertrophic signaling and increase the probability of arrhythmic events. Recent work on transgenic mice containing troponin mutations has provided evidence of these outcomes, although whether this is due to primary effects of the mutant protein or compensatory changes is unclear. Some studies have shown profound increases in basal [Ca^2+^]*_i_* in the presence of increased Ca^2+^ buffering (13, 14), whereas others have found the opposite effect depending on the age of the mice (15, 16). In this study we have systematically tested the changes to both Ca^2+^ cycling and Ca^2+^-dependent signaling in a stable but short-term cardiomyocyte model of HCM. This approach allows evaluation of the direct cellular consequences of a HCM mutation, free of the secondary effects of pathological remodeling caused by compensatory (or maladaptive) gene expression. For example, reduction of SERCA levels (17) and myofilament protein isoform switching (18), both well known hallmarks of heart failure and cardiomyopathy, would be expected to confound electrophysiological and contractile changes caused by the primary mutation in animal models and patients. We have used guinea pig left ventricular cardiomyocytes to model human cardiomyocyte Ca^2+^ cycling more accurately than previous studies published in transgenic mice. Mouse cardiomyocytes fundamentally differ from both human and guinea pig in structure and function. For example, they contain predominantly fast α-MyHC *versus* slow β-MyHC in human and guinea pig (19). The generation of Ca^2+^ transients relies almost entirely on Ca^2+^-induced Ca^2+^ release from the SR with very little contribution from NCX current, whereas in humans and guinea pigs, the NCX contribution is ∼30% (20). Cardiac action potentials in mice lack any appreciable plateau and differ markedly in waveform (21), indicating a restructured electrophysiological regulation.

We have engineered adenoviruses to express WT and R92Q cTnT, WT, and R145G cTnI, and WT and D175N α-TM. By measuring the impact of mutations affecting three different sarcomeric proteins, we aimed to identify hallmark changes in Ca^2+^ handling in HCM. Furthermore, we present for the first time a novel analytical paradigm to fully incorporate the consequences of Ca^2+^ buffering on cardiomyocyte Ca^2+^ transients and Ca^2+^ handling protein activities. We consider the dynamics of total intracellular Ca^2+^ ([Ca^2+^]_total_) derived from NCX integral measurements rather than free Ca^2+^ ([Ca^2+^]*_i_*) from fura2 fluorescence when assessing SR load, fractional SR release, and SERCA activity. When applied, our data show that the primary effects of HCM mutant gene expression include altered Ca^2+^ transients and increased myofilament Ca^2+^ buffering, increased [Ca^2+^] in both the SR and cytoplasm, and activation of Ca^2+^-dependent signaling mediated by Ca^2+^/calmodulin-dependent protein kinase II (CaMKII), calcineurin/nuclear factor of activated T-cells (NFAT), and mitogen-activated protein kinase/ERK.

## Results

### Adenoviral expression of human troponin and tropomyosin in guinea pig cardiomyocytes

**Figure 1.**
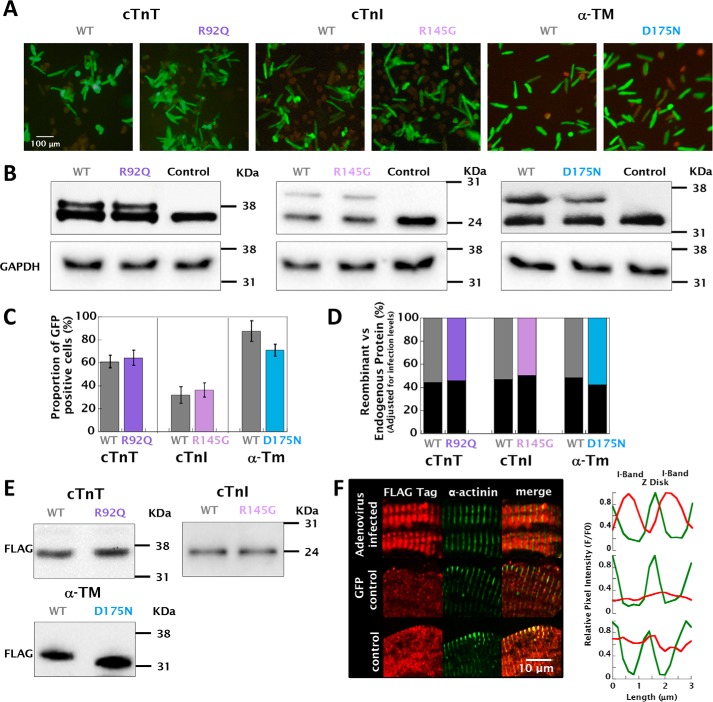
**Recombinant adenoviral infection, protein expression, and localization in guinea pig left ventricular cardiomyocytes.** *A* shows the representative GFP expression in cardiomyocytes infected with recombinant adenovirus at ∼1000 MOI 48 h after transfection. *B* shows the relative protein expression of FLAG-tagged human recombinant WT/R92Q cTnT, WT/R145G cTnI, and WT/D175N α-TM compared with endogenous guinea pig subunits probed using anti-cTnT, anti-cTnI, and anti-α-TM primary antibodies, respectively. The *third lane* of each gel in *B* shows that endogenous protein in uninfected control cardiomyocytes is higher than endogenous levels from infected cardiomyocytes, therefore suggesting that recombinant protein replaces the endogenous at the myofilament. The *bar graph* in *C* shows the relative infection ratio of each virus as a percentage of green cells, whereas *D* shows the relative expression levels of endogenous (*dark gray bars*) *versus* recombinant (*colored bars*) protein, calculated from densitometery measurements of doublet bands in *B* multiplied by the relative expression levels of each virus calculated in *C. E* shows Western blots for cell lysates expressing each different recombinant protein using anti-FLAG tag primary antibody. *F* shows a magnified image of adenovirally expressed FLAG-tagged protein localized to the I-band in cardiomyocytes. Colocalization used an anti-FLAG tag primary antibody (conjugated to Alexa 568, *red*), with counterstain provided using an α-actinin antibody (conjugated to Alexa 633, false colored *green*) to stain the z disks. Colocalization was confirmed in the adjacent intensity profile plots. I-band staining usually presents as a doublet; however, because of the contracted nature of isolated cells, the signal from the I-band appeared as a single band with the limited resolution provided by the confocal microscope. (A full breakdown of the localization of each recombinant protein used can be found in Fig. S2.)

Isolated guinea pig left ventricular cardiomyocytes were infected with ∼1000 MOI of recombinant adenovirus. The level of infection was between 32.1 ± 7.2 (WT TnI) and 87.6 ± 9.1% (WT TM), calculated from the coexpression of human recombinant GFP (Fig. 1, *A* and *C*). The relative expression of thin filament regulatory protein subunits was determined by Western blotting and subsequently adjusted by the infection levels for each cell preparation tested. Conjugation of an N-terminal (cTnT and cTnI) or C-terminal (α-TM) FLAG tag increased the molecular weight of the recombinant proteins sufficiently to allow differentiation from the endogenous subunit (Fig. 1*E*). The relative expression level of recombinant protein in purified cardiomyocytes coexpressing human recombinant GFP was found to be 54.3 ± 9.5% (cTnT R92Q), 49.3 ± 8.5% (cTnI R145G), and 57.6 ± 7.5% (TM D175N) (Fig. 1, *B* and *D*), thus likely to closely reflect the levels of mutant protein found in patients with autosomal dominant cardiomyopathy. The recombinant FLAG-tagged thin filament proteins were localized to the I-band of the myofilaments of infected cardiomyocytes (Fig. 1*F* and Fig. S2). To assess the impact of culture time on cardiomyocyte function, we compared the contractility and Ca^2+^ transients of cardiomyocytes cultured for 1, 24, and 48 h along with the extent of t-tubular dedifferentiation (Fig. S3). We observed a small but significant reduction in the number of t-tubules and a prolongation in the relaxation and Ca^2+^ reuptake after 48 h (Fig. S4 and Table S1).

### The presence of the Ca^2+^-sensitizing mutations increases myofilament Ca^2+^ buffering and drives altered Ca^2+^ homeostasis

**Figure 2.**
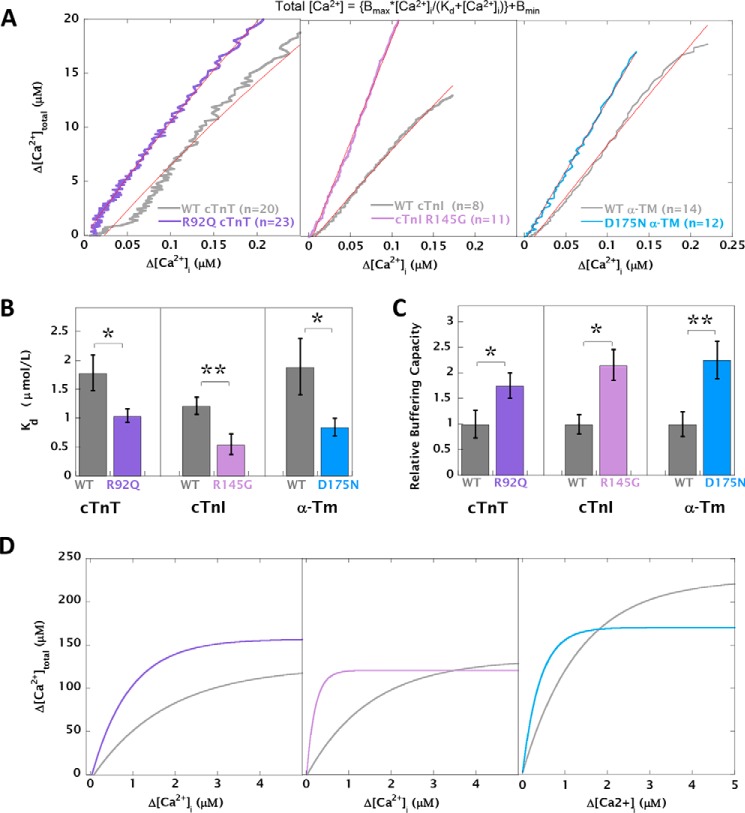
**HCM mutations acutely decrease the apparent *K_d_* of cytosolic Ca^2+^ buffering.** Cardiomyocytes expressing WT or mutant cTnT/cTnI/α-TM were simultaneously assessed to measure [Ca^2+^]*_i_* by fura2 fluorescence and NCX current (to calculate [Ca^2+^]_total_) by voltage clamping upon the rapid application of 10 mm caffeine. *A*, plots of Δ[Ca^2+^]*_i_ versus* Δ[Ca^2+^]_total_ for cardiomyocytes containing WT cTnT (*n* = 20) *versus* R92Q cTnT (*n* = 23), WT cTnI (*n* = 9) *versus* R145G cTnI (*n* = 11), and WT α-TM (*n* = 12) *versus* D175N α-TM (*n* = 14) reveal increased buffering capacity in cells containing HCM mutants. The *red lines* show best fits to the following: [Ca^2+^]_total_ = {*B*_max_*[Ca^2+^]*_i_*/(*K_d_* + [Ca^2+^]*_i_*)} + *B*_min_. *B*, bar graph comparing mean *K_d_* values for the fits calculated in *A. C*, *bar graph* showing the relative estimated changes in buffering capacity (calculated from relative *K_d_* and *B*_max_ values for individual cells undergoing the buffing protocol), where mutant calls have been compared with WT-infected cells set to 1. *D*, cytosolic buffering *curves* over a wide range of Ca^2+^ concentration drawn using the *K_d_* and *B*_max_ values calculated in *A*. Comparison of WT with HCM mutant: **, *p* < 0.01; *, *p* < 0.05.

[Ca^2+^]_total_ and [Ca^2+^]*_i_* were measured upon the application of 10 mm caffeine using simultaneous measurement of fura2 fluorescence ratio and whole-cell voltage-clamp current in a method adapted from Trafford *et al.* (22) (Fig. S6). We postulated that increasing the Ca^2+^ affinity of myofilaments by introducing a Ca^2+^ sensitizing HCM mutation will in turn increase total Ca^2+^ buffering in the intact cardiomyocytes. We fitted the calibrated total *versus* free [Ca^2+^]*_i_* data to a Michaelis–Menten type equation (Fig. 2*A*). The *K_d_* of Ca^2+^ buffering was significantly decreased in the presence of each HCM-causing mutation (Fig. 2*B* and Table S2). However, the total Ca^2+^ occupancy of the myofilaments (*B*_max_) was not significantly altered between groups (Table S2). The relative buffering at low [Ca^2+^] is described by B_max_/*K_d_*, and this ratio was increased for all three mutants compared with WT (Fig. 2*C*). The calculated relationship between [Ca^2+^]_total_ and [Ca^2+^]*_i_* over the full range of [Ca^2+^] is shown in Fig. 2*D*. We have used these fits to convert observed [Ca^2+^]_free_ to [Ca^2+^]_total_ for each cell and calculate changes in SR load and SERCA activity. This incorporates the effect of altered Ca^2+^ buffering when considering these key Ca^2+^-handling parameters.

### Cardiomyocytes expressing Ca^2+^-buffering HCM-causing mutations exhibit altered Ca^2+^ transients and contractility

**Figure 3.**
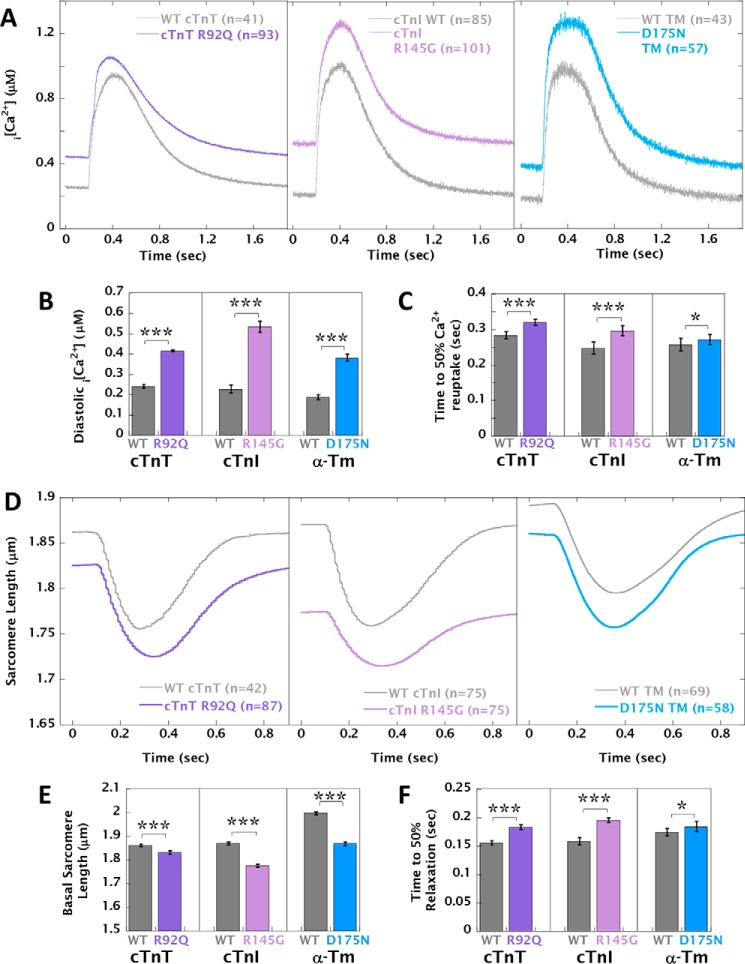
**Unloaded sarcomere shortening and Ca^2+^ transient measurements in adenovirally infected guinea pig left ventricular cardiomyocytes.** *A*, intracellular Ca^2+^ transients of cardiomyocytes loaded with 1 mm fura2 at 0.5 Hz pacing, expressing HCM-causing mutations cTnT R92Q, cTnI R145G, and α-TM D175N compared with cardiomyocytes infected with the equivalent WT protein. *B* and *C*, the average diastolic [Ca^2+^]*_i_* and time to 50% Ca^2+^ reuptake, respectively. *D*, the corresponding unloaded sarcomere shortening measurements at 1-Hz pacing. *E* and *F*, the average basal sarcomere length and time to 50% relaxation, respectively. The pairwise sarcomere shortening of fura2-loaded cardiomyocytes acquired at 0.5 Hz is shown in Fig. S7. Each *curve* was averaged from multiple cells (*n*) taken from at least four separate cell preparations; total *n* numbers are given in the legends of each plot. Comparison of WT with HCM mutant: ***, *p* < 0.001; *, *p* < 0.05.

In analyses of cytosolic [Ca^2+^]*_i_* transients at 0.5-Hz pacing (Fig. 3*A*), cells expressing HCM-causing mutations had significantly higher diastolic [Ca^2+^]*_i_* (Δ[Ca^2+^]*_i_* = 0.175 ± 0.007 to 0.307 ± 0.011 μm) (Fig. 3*B* and Table S3*A*) and significantly prolonged rates of Ca^2+^ reuptake (Δτ = 0.027 ± 0.003 to 0.041 ± 0.002 s^−1^) (Fig. 3*C* and Table S3*A*) compared with cardiomyocytes expressing the corresponding WT protein. The time to 50% Ca^2+^ release from the SR and the Ca^2+^ transient amplitude were not significantly different between groups. In measurements of sarcomere shortening at 1-Hz pacing, all three HCM mutations were found to decrease basal sarcomere length compared with WT (ΔSL = −0.030 ± 0.007 to −0.094 ± 0.007 μm) in line with the higher levels of diastolic [Ca^2+^]*_i_*. Cardiomyocytes expressing HCM mutations also had significantly prolonged relaxation (Δ*T*_50_ = 0.011 ± 0.002 to 0.037 ± 0.003 s) (Fig. 3, *D–F*, and Table S3*B*). Cardiomyocytes expressing the R145G mutation in cTnI displayed reduced fractional shortening compared with WT; these cells also had the shortest diastolic sarcomere length (1.776 ± 0.007 *versus* 1.865 ± 0.009 μm in WT), and this may impose some limit on the extent of shortening compared with the other two mutations, because of fura2 accumulation (23) and resultant Ca^2+^ chelation in our loaded cardiomyocytes. The phenotype conferred by HCM mutations in fura2-loaded cardiomyocytes at 0.5 Hz was qualitatively the same, despite reduced fractional shortening caused by the presence of the chemical dye (Fig. S7 and Table S3*C*).

### Increased myofilament Ca^2+^ buffering results in higher peak NCX current, SR load, and RyR receptor leak/load relationship along with activation of CaMKII signaling

**Figure 4.**
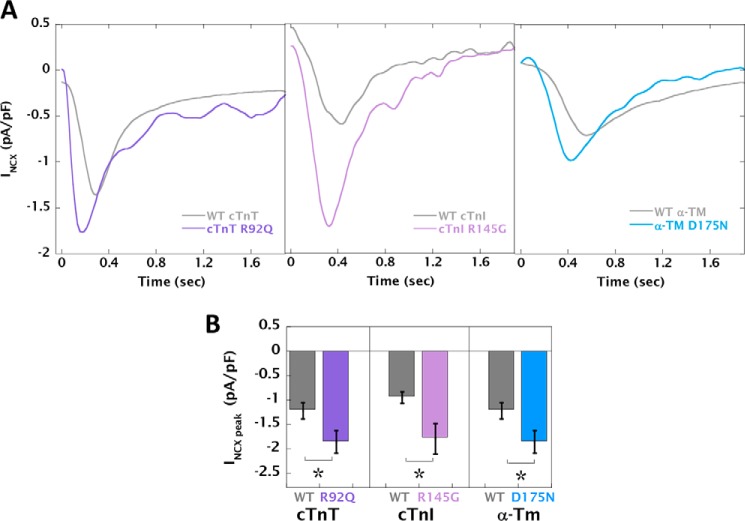
**HCM mutant-infected cardiomyocytes have increased NCX current.** *A*, representative traces from voltage-clamp recordings during the application of 10 mm caffeine. *B*, the average peak amplitude for WT cTnT (*n* = 24), cTnT R92Q (*n* = 23), WT cTnI (*n* = 8), cTnI R145G (*n* = 8), WT α-TM (*n* = 15), and α-TM D175N (*n* = 19). Comparison of WT with HCM mutant: *, *p* < 0.05.

The NCX current was measured during the direct application of 10 mm caffeine by whole-cell voltage-clamp recordings (Fig. S6). The peak *I*_NCX_ current was significantly increased in cardiomyocytes expressing the HCM mutants. (Fig. 4, *A* and *B*, and Table S4). Simultaneous measurement of L-type Ca^2+^ current showed no difference between WT and HCM mutant cardiomyocytes (Fig. S8 and Table S4).

**Figure 5.**
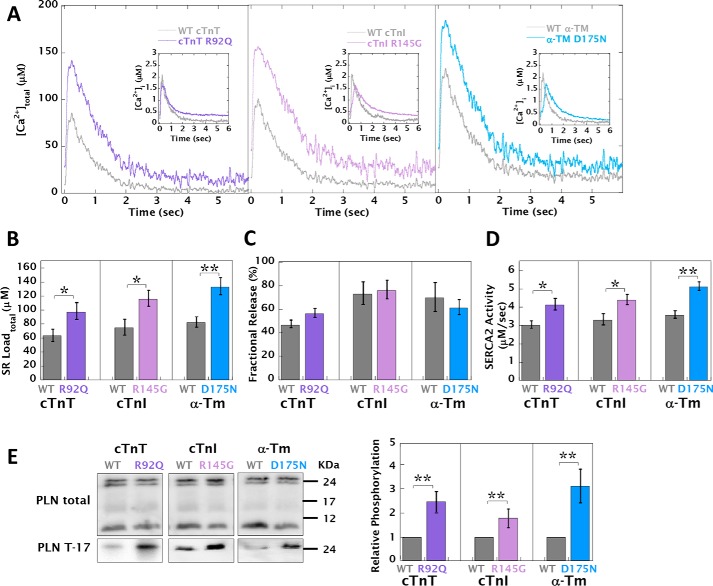
**HCM mutant-infected cardiomyocytes have increased SR load, fractional SR Ca^2+^ release, and SERCA2 activity via CaMKII-dependent PLN phosphorylation.** *A*, representative caffeine-induced [Ca^2+^]_total_ transients generated by the direct perfusion of 10 mm caffeine after a 5-s pause of pacing comparing WT and HCM mutant-transfected cardiomyocytes, where each transient amplitude has been adjusted from [Ca^2+^]*_i_* (*inset*) to [Ca^2+^]_total_ using the buffering curves in Fig. 1*A*. Bar graphs show the SR load of [Ca^2+^]_total_ (*B*), fractional release of Ca^2+^ (calculated from the [Ca^2+^]_total_ caffeine transient amplitudes in *B* and Table S5*B* subtracted from the preceding [Ca^2+^]_total_ transient amplitude shown in Table S5*B*) (*C*), and the SERCA2 activity (calculated by the subtraction of [Ca^2+^]_total_ τ-decay constants (total) from the [Ca^2+^]_total_ caffeine transient (NCX) τ-decay rates Table S5*B*) (*D*). The extracted parameters from [Ca^2+^]*_i_* baseline and caffeine transients are also tabulated in Table S5*A*. *E*, representative Western blots of total and phosphothreonine 17 PLN. The *bar graph* shows the densitometric quantification of phosphorylation increase (*n* = 5). All preparations were paced for 8 h at 0.5 Hz. **, *p* < 0.01; *, *p* < 0.05; *ns*, *p* > 0.05.

Using a standard method (24) that utilizes the fura2-based measurement of caffeine [Ca^2+^]*_i_* transients, SR load, fractional SR Ca^2+^ release, and SERCA activity were calculated and found to be decreased, increased, and unchanged, respectively (Fig. 5*A*, *insets*, and Table S5*A*). Furthermore, we found that these alterations were independent of pause duration prior to caffeine spritz (Table S6). However, we also calculated caffeine and Ca^2+^ transients expressed as [Ca^2+^]_total_ derived from the NCX current integral, thus incorporating any alterations to Ca^2+^ buffering, and used this approach to recalculate SR load, fractional SR Ca^2+^ release and SERCA activity (Fig. 5*A* and Table S5*B*). HCM mutant cells consistently displayed a higher SR load (Δ[Ca^2+^]_total_ = 34.1 ± 3.2 to 51.3 ± 8.2 μm) (Fig. 5*B*). The preceding [Ca^2+^]*_i_* transient amplitude (Table S5*A*) was also converted to [Ca^2+^]_total_ (Table S5*B*) to calculate the fractional SR Ca^2+^ release and SERCA2 activity. HCM cells had unchanged SR fractional release compared with WT (Fig. 5*C*) and had increased SERCA2 activity (Δrate = 1.53 ± 0.30 to 3.87 ± 0.42 s^−1^) (Fig. 5*D*).

The increases in SR load and SERCA2 activity appear to be driven by increased CaMKII-dependent phosphorylation of phospholamban (PLN). Relative phosphothreonine 17 PLN levels were increased in HCM cells (between 87 ± 27 and 220 ± 72% more than WT) (Fig. 5*E*), whereas the PKA site at serine 16 of PLN and absolute levels of NCX and SERCA2 remained unaltered (Fig. S9). This result also confirms the need to consider the [Ca^2+^]_total_ transient amplitudes when investigating SERCA2 activity and SR load in cardiomyocytes with altered Ca^2+^ buffering.

**Figure 6.**
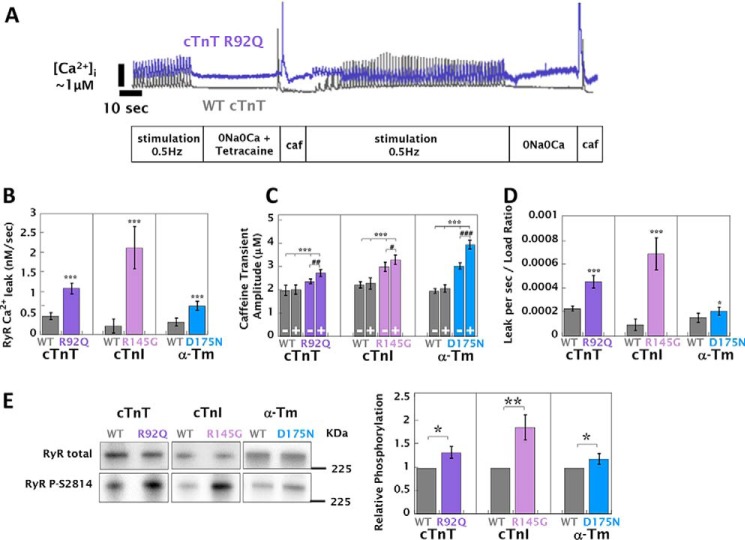
**HCM mutant-infected cardiomyocytes have increased RyR leak/load relationships and CAMKII activation of RyR.** SR Ca^2+^ load and Ca^2+^ leak via the RyR were assessed by the sequential perfusion of 1.8 mm CaCl_2_ (*A*), Na^+^- and Ca^2+^-free solution containing 1 mm tetracaine for 50 s (*B*), the direct application of 10 mm caffeine (*C*), 1.8 mm CaCl_2_ for 100 s (*D*), Na^+^- and Ca^2+^-free solution for 50 s (*E*), and a direct application of 10 mm caffeine (*F*). *A*, representative experimental traces for WT cTnT (*gray*) and cTnT R92Q (*purple*) cardiomyocytes. All mutants tested were analogous to cTnT R92Q, whereas all control and WT-infected cells tested resembled WT cTnT. *B*, the observed RyR-dependent leak rate. *C*, the buffering-adjusted caffeine transient amplitude taken after perfusion with either 0Ca0Na (−) or with 0Na0Ca solution containing 1 μm tetracaine (+). *D*, the leak/load ratio, calculated as the leak rate divided by the caffeine transient amplitude at the end of each experiment. Each *bar graph* is an average of 30 cells, 15 of which were acquired as shown in *A* or *B*, whereas a further 15 were acquired by reversing the sequence of 0Na0Ca solution with or without 1 μm tetracaine to prevent errors from fura2 signal degradation and cell fatigue. A full breakdown of Δ[Ca^2+^]*_i_* for each perfusion switch can be found in Fig. S10, and the extracted parameters are also tabulated in Table S7. Representative Western blots of total and phosphoserine 2814 RyR are given in *E*, with the adjacent *bar graph* showing the average change in phosphorylation from densitometry measurements, all preparations were paced for 8 h at 0.5 Hz. Unpaced preparations showed no significant changes at the same site (data not shown). ***, *p* < 0.001; **, *p* < 0.01; *, *p* < 0.05 for comparing WT to HCM mutant. ###, *p* < 0.001; ##, *p* < 0.01; #, *p* < 0.05 for comparing the presence or absence of 1 mm tetracaine.

Increased SR load and diastolic [Ca^2+^]*_i_* in HCM cardiomyocytes suggest there may be Ca^2+^ leak from the RyR. To test this, we used a method adapted from Shannon *et al.* (25) (Fig. 6*A*) and found that RyR leak is profoundly increased in cells expressing HCM mutations (Fig. 6*B*). It was also observed that RyR blockade with tetracaine increased the [Ca^2+^]*_i_* caffeine transient amplitude by 12–25% in HCM cells (Fig. 6*C*) but had no effect on WT-infected or uninfected control myocytes. Despite the combination of increased leak (Fig. 6*B*) and [Ca^2+^]*_i_* SR load (adjusted for myofilament buffering calculated in Fig. 2*C*) (Fig. 6*C*), the relative leak load relationship was still higher in HCM cardiomyocytes (Δμm/s/μm = 5.24 × 10^−5^ ± 1.65 × 10^−5^ to 2.22 × 10^−4^ ± 3.75 × 10^−5^) (Fig. 6*D*), suggesting that other factors may be driving RyR channel open probability. We measured the phosphorylation status of each of the known regulatory sites on the RyR (serine 2030, 2808, and 2814) and found that (consistent with changes in PLN phosphorylation), there was higher phosphorylation at the RyR CaMKII site serine 2814 (20 ± 11 to 87 ± 27% greater than WT) (Fig. 6*E*); phosphorylation at the PKA/PKG sites (serine 2030 and 2808) did not differ between WT and HCM mutant cardiomyocytes (Fig. S9).

### Altered Ca^2+^ homeostasis in HCM mutant cardiomyocytes activates NFAT and ERK signaling

**Figure 7.**
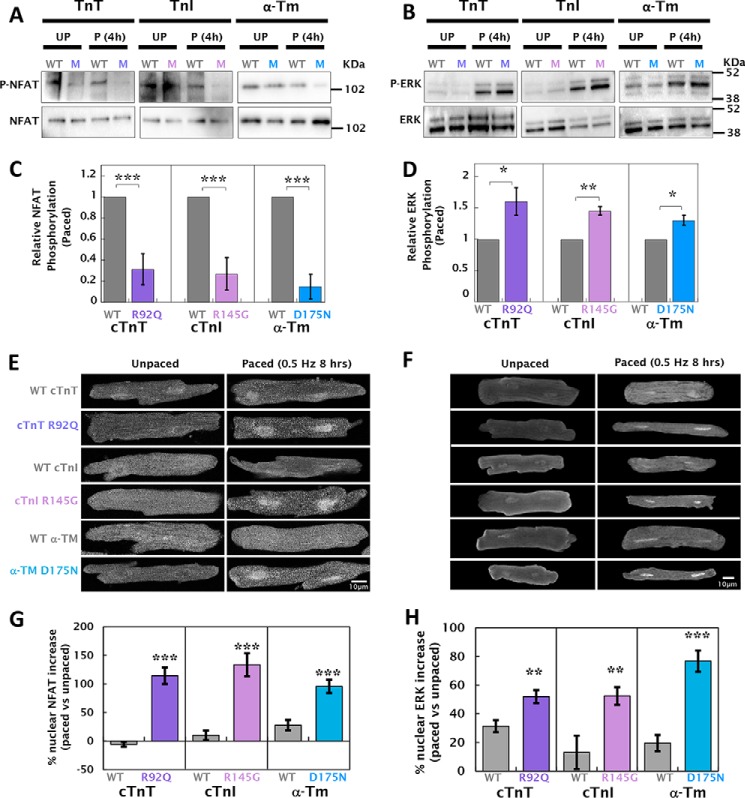
**HCM mutant-infected cardiomyocytes have altered NFAT and ERK phosphorylation resulting in increased nuclear localization.** *A*, total NFAT and phosphoserine 165 NFAT were measured by Western blotting of transfected cardiomyocytes either paced at 0.5 Hz for 8 h (*P*) or unpaced (*UP*). *B*, densitometric quantification of blots from paced cells indicates that expression of HCM mutations results in a significant decrease in phospho-NFAT compared with WT (*n* = 6). Similarly, *C*, ERK phosphorylated at threonine 202/tyrosine 204 and total ERK were measured by Western blotting of transfected cardiomyocytes either paced or unpaced. *D*, densitometric quantification of blots from paced cells show that expression of HCM mutations results in a significant increase in phospho-ERK compared with WT (*n* = 6). Relative changes in phosphorylation of NFAT and ERK in unpaced cardiomyocytes and the percentages of change in phosphorylation caused by pacing for each group are tabulated in Table S8. *E* and *F* show representative immunofluorescence images using anti-NFAT and anti-ERK antibodies, respectively. Individual panels compare HCM mutant to WT-infected cardiomyocytes either unpaced or paced at 0.5 Hz for 8 h. *G* and *H*, percentage of change in NFAT or ERK nuclear localization between paced and unpaced cardiomyocytes for each mutant and WT-infected group (*n* = 30–40 cells in each group). ***, *p* < 0.001; **, *p* < 0.01; *, *p* < 0.05; *ns*, *p* > 0.05. Normalized relative localization *versus* background cytosolic NFAT and ERK comparing WT *versus* mutant measurements are tabulated in Table S8.

Ca^2+^-dependent signaling was assessed by the phosphorylation status and immunolocalization of the NFAT and ERK, two key mediators of HCM pathogenesis (26). Fig. 7 (*A* and *B*) show that both NFAT and ERK phosphorylation (NFAT at serine 165 and ERK at threonine 202/tyrosine 204) are altered in paced cardiomyocytes containing HCM mutations. NFAT-c3 phosphorylation is unchanged in unpaced cells but is substantially dephosphorylated in paced (0.5 Hz for 8 h) HCM mutant cardiomyocytes (phosphorylation reduced by 68 ± 15 to 85 ± 12% compared with WT) (Fig. 7*C*). ERK phosphorylation is increased in WT cells upon pacing but significantly more in the presence of HCM mutations (30 ± 8 to 60 ± 22% greater than WT) (Fig. 7*D*). Concordant nuclear translocation of NFAT-c3 and ERK is shown in Fig. 7 (*E* and *F*). In the absence of pacing, the distribution of NFAT-c3 was equal between the nucleus and cytosol in both mutant and WT cells (Table S8). However, the presence of a Ca^2+^-sensitizing HCM mutation in cells that had been paced for 8 h prior to fixation caused a 95.6 ± 11.6 to 133.6 ± 20.2% increase in the nuclear localization of NFAT-c3 (Fig. 7*G*). Pacing of WT cardiomyocytes resulted in a 13.1 ± 11.6 to 31.4 ± 4.2% increase in nuclear ERK; however, HCM cardiomyocytes had significantly higher nuclear translocation (52.1 ± 4.5 to 76.8 ± 7.4%) (Fig. 7*H* and Table S8).

## Discussion

This study set out to investigate the effects of Ca^2+^-sensitizing HCM mutations in thin filament regulatory proteins on myofilament Ca^2+^ buffering and the resultant consequences to intracellular Ca^2+^ handling and hypertrophic signaling. We examined mutants in three different regulatory proteins in adult guinea pig cardiomyocytes transfected with recombinant adenovirus, which resulted in ∼50% incorporation of the mutant protein. Mutations in troponin and tropomyosin only comprise ∼10% of all mutations found in HCM patients (27); however, they affect contractile regulation in a similar way to the more common mutations in βMyHC and MyBPC (3). The selected mutations cTnT R92Q, cTnI R145G, and α-TM D175N are also among the most prevalent in each gene and have similar HCM phenotypes (28, 29). Characterization of the infected cardiomyocytes showed that the presence of a Ca^2+^-sensitizing thin filament mutant doubled the Ca^2+^ buffering of the myofilament and had a profound effect on Ca^2+^ handling. These changes were consistent among the disease genes with the cTnI R145G mutation having the largest effect in many of the assays. These functional alterations are likely to be maintained or even accentuated by the increased CaMKII-dependent phosphorylation of Ca^2+^ handling proteins (PLN and RyR). We also show the direct link between altered intracellular Ca^2+^ handling and activation of key regulators of cardiac hypertrophy. Both NFAT and ERK are translocated to the nucleus as a result of Ca^2+^ dysregulation in cardiomyocytes containing HCM mutations. These data emphasize the intimate link between myofilament Ca^2+^ buffering, Ca^2+^ handling, and the initiation of Ca^2+^-mediated hypertrophic signaling.

Our study builds on and extends earlier work, much of it using cardiomyocytes isolated from mouse models of HCM. Our experimental design provides the strongest evidence to date that altered Ca^2+^ buffering can be directly attributed to the primary effect of a mutation on myofilament Ca^2+^ affinity, because of the short-term transfection model used. Previous detailed electrophysiological characterization of HCM cardiomyocytes used transgenic mice in which the disease-causing mutation is expressed from birth or earlier, and thus the observed functional changes reflect a mixture of primary effects caused by the mutation and secondary compensatory alterations caused by molecular and (in longer-term studies) physiological remodeling. Furthermore, we opted to use adult guinea pig cardiomyocytes in which the myosin isoform and Ca^2+^ handling more closely resemble that found in human. Murine cardiomyocytes contain principally α-MyHC that has faster enzyme kinetics than the β isoform, which predominates in both guinea pig and human cardiomyocytes (19), and has been shown to be an important determinant when assessing myocyte or cardiac function (30). Also, in mice, Ca^2+^ reuptake during diastole is almost entirely dependent on SERCA2, whereas in humans and guinea pigs, the sarcolemmal NCX makes a substantial contribution (20). This and the shorter action potential lacking an appreciable plateau (21) may make the guinea pig a more accurate model for determining the effects of altered myofilament Ca^2+^ affinity on Ca^2+^ handling in HCM patients.

Some recent work analyzing Ca^2+^ handling in transgenic mice containing Ca^2+^-sensitizing mutations has shown profound increases in basal [Ca^2+^]*_i_* in the presence of increased Ca^2+^ buffering (13, 14), whereas others have found the opposite effect depending on the age of the mice (15, 16). One of the former experiments also shows a pause-dependent increase in SR load when HCM variants are present (13); however, using guinea pig cardiomyocytes, which have slower Ca^2+^ cycling, alterations to SR Ca^2+^ are seen independently of pausing prior to caffeine application. In the latter cases there are profound alterations in the levels of Ca^2+^-handling proteins, such as SERCA and PLN, and age-dependent alterations to CAMKII phosphorylation levels. These findings are in agreement with our shorter-term study where Ca^2+^ buffering appears to directly affect SERCA2 activity via CaMKII activation and subsequent phosphorylation of PLN threonine 17. Adenoviral-mediated transfection of HCM mutations has previously been carried out using rat primary cardiomyocytes. The restrictive cardiomyopathy R193H TnI mutant increased myofilament Ca^2+^ sensitivity and decreased sarcomere length, as well as increasing both relaxation time and Ca^2+^ transient decay (31). Of note, cardiomyocytes isolated from a transgenic mouse model of the same mutant showed prolonged relaxation with no change in Ca^2+^ transient decay, suggesting secondary changes at the level of Ca^2+^ handling (32).

The observed increase in diastolic Ca^2+^ is likely to be caused by the slower release of Ca^2+^ from the myofilament during Ca^2+^ reuptake by SERCA and maintained by increased RyR leak. Isolated cardiomyocytes from myectomy samples taken from HCM patients with different genetic mutations showed increases in diastolic Ca^2+^ (33) analogous to those in our study. This may indicate that the primary defects in Ca^2+^ handling may again begin to predominate as disease pathogenesis progresses to end stage and the chronically remodeled myocardium begins to fail. More recent work from human samples shows that CaMKII signaling and functional effects on SERCA/PLN are preserved in end stage HCM; interestingly the study also shows compensatory changes of absolute SERCA levels (34). In contrast with our findings, RyR phosphorylation was unchanged in cardiomyocytes from 10 patients. This suggests that some aspects of Ca^2+^ buffering in HCM are preserved throughout the natural history of the disease in nonmurine HCM, whereas others are compensated for to preserve long-term myocyte function during disease progression. It has also been suggested that CaMKII plays a nodal role in intracellular signaling (35); our study confirms that CaMKII mediates phosphorylation of both PLN and RyR, and its activity is increased in HCM cardiomyocytes. Longer-term studies suggest secondary changes, potentially under the control of mechanochemotransduction pathways such as nitric oxide, which may shut off some of these nodes (35, 36), because the myocardium attempts to achieve a homeostatic equilibrium of the Ca^2+^ pool to preserve contractile function.

Work on both human tissue and transgenic animals shows conflicting results when assessing SR Ca^2+^, NCX, and SERCA2 activity (13–15). This is the first study to directly account for the myofilament buffering when calculating these parameters. Increased myofilament Ca^2+^ occupancy will mask recordings that rely on cytoplasmic fura2 fluorescence, leading to under representations of the [Ca^2+^] used to estimate NCX and SERCA2 activity. If the necessary adjustments for buffering are made (as in this study), a paradigm of Ca^2+^ overload with altered Ca^2+^ compartmentalization begins to develop. There is a need for greater mechanistic insight of subdomain Ca^2+^ in HCM. The emerging next generation of genetically encoded Ca^2+^ sensors (GECOs) are now sensitive and fast enough kinetically to be used in the cardiomyocyte (37). They can be cross-linked to a range of proteins across the contracting cardiomyocyte and dynamically signal changes in Ca^2+^ microdomains (38).

We show that NFAT and ERK are activated in HCM infected cardiomyocytes when paced in culture, in agreement with the mechanisms proposed by Davis *et al.* (39). Here we show that Ca^2+^ handling directly facilitates NFAT dephosphorylation, leading to nuclear translocation in addition to CaMKII activation, which differs from findings in neonatal rat cardiomyocytes, where the opposite effect is seen (40). However, we also describe acute ERK phosphorylation and nuclear translocation in HCM in the absence of pacing. This suggests a primary mechanism distinct from that of NFAT driving these changes. Microdomain Ca^2+^ or indirect HCM mutation effects caused by altered contractility or energetics may underlie these observations.

Our work and that of others help to establish a paradigm of Ca^2+^-dependent myocardial remodeling in HCM: the underlying genetic defect causes increased myofilament Ca^2+^ buffering and altered Ca^2+^ handling. Ca^2+^-dependent downstream signaling cascades may then drive deleterious cellular remodeling. Other proposed mechanisms include energetic compromise from inefficient ATP utilization affecting the metabolic milieu of the cardiomyocyte and impairing energy-requiring ionic homeostasis, especially Ca^2+^ reuptake by SERCA2 (3, 41). Thus, both direct and indirect alterations in Ca^2+^ handling may work in concert to generate the macroscopic HCM disease phenotype. Furthermore previous studies have implicated myocyte disarray and interstitial fibrosis as the main propagators of ventricular arrhythmias in HCM pathophysiology (42). Our findings re-enforces the view that altered intracellular Ca^2+^ homeostasis in the diseased cardiomyocyte, brought about as a direct consequence by the primary gene mutation, may be important in arrhythmic events observed in HCM patients.

These data raise the possibility that correction of Ca^2+^ dysregulation and signaling could be disease-modifying in HCM and improve outcomes. Direct targeting of myofilament Ca^2+^ sensitivity provides the most attractive potential therapeutic approach. Small molecules such as the green tea polyphenol epigallocatechin-3-gallate have been shown to bind to cTnC (43) and desensitize the myofilament (44), although it should be noted that epigallocatechin-3-gallate currently lacks specificity and potency to be useful in itself (45). Such approaches using derivative compounds may provide a tractable method for drug treatment to prevent or even regress HCM disease pathology by targeting its primary cause.

## Experimental procedures

### Adenoviral design and production

Adenoviruses were engineered to contain either WT or HCM mutant FLAG-tagged thin filament proteins (cTnT R92Q, cTnI R145G, and α-TM D175N) using the AdEasy XL viral production system (Agilent Technologies). Viral particles were purified by CsCl gradient centrifugation and desalted by dialysis, and the number of plaque-forming units per ml was determined using the manufacturer's standard protocols.

### Isolation of guinea pig left ventricular cardiomyocytes

This investigation was approved by the Animal Welfare and Ethical Review Board at the University of Oxford and conforms to the UK Animals (Scientific Procedures) Act, 1986, incorporating Directive 2010/63/EU of the European Parliament. Left ventricular cardiomyocytes were isolated from guinea pig heart, by standard collagenase perfusion and mechanical agitation (46). Cardiomyocytes were incubated in ACCITT_3_ culture medium (47) at 37 °C and 5% CO_2_ in the presence of ∼1000 MOI of adenovirus for 48 h. All subsequent functional experiments were carefully controlled for culture time, whereas viral MOI ratios were assessed throughout the duration of the study to ensure that the validity of the model was maintained. All experiments detailed herein compare HCM mutant-infected cardiomyocytes with similarly infected cardiomyocytes expressing human FLAG-tagged recombinant WT protein. Uninfected control cardiomyocytes broadly resembled the WT-infected controls; any exception to this is detailed in Tables S2–S7.

### Determination of optimum fura2-AM-ester loading concentration

Optimum fura2-AM-ester loading in isolated cardiomyocytes was determined by signal-to-noise analysis using Ionwizard software (IonOptix). 100,000–150,000 viable guinea pig left ventricular cardiomyocytes were incubated with 5, 1, 0.5, or 0.1 μm fura2-AM ester (Life Technologies) in buffer containing 150 mm NaCl, 10 mm HEPES, 7 mm glucose, 1 mm MgCl, 1 mm KCl, 0.3 mm NaH_2_PO_3_, and 250 μm CaCl_2_, pH 7.4, with NaOH. F365/380 was determined using IonOptix μstep under electrical pacing at 40 V and 0.5 Hz to establish basal noise and peak stimulated fluorescence conditions. It was determined that 1 μm was the minimum concentration/time required to give a dynamic Ca^2+^ signal (with lower concentrations giving signal to noise ratios of 1 or lower) and was therefore used in all subsequent experiments (Fig. S5).

### Measurement of Ca^2+^ buffering, NCX current, SR content, and SERCA activity

Cardiomyocytes were loaded with fura2, attached to a whole-cell voltage-clamp pipette, and spritzed with 10 mm caffeine. Intracellular Ca^2+^ buffering was calculated from the simultaneous measurement of total and free Ca^2+^ ([Ca^2+^]_total_ and [Ca^2+^]*_i_*, respectively) using a previously reported technique (22). Specifically, caffeine caused the release of Ca^2+^ from SR and induced a brief Ca^2+^ transient measured using fura2 (“caffeine transient”), as well as Ca^2+^ efflux via NCX (NCX current), allowing [Ca^2+^]*_i_* and [Ca^2+^]_total_ to be calculated. To exclude the other flux mechanism's contribution to the extrusion of Ca, the measured NCX currents were integrated and corrected as previously described (48). After this correction, we now assume that extrusion of Ca^2+^ released from SR by caffeine is only via NCX. Based on this foundation, we then determined (at two time points) the change of integral of NCX current. This gave the Δ[Ca^2+^]_total_, whereas the change of intracellular Ca^2+^ transient measured by fura2 gave Δ[Ca^2+^]*_i_* during caffeine application. Thus, the relationship between Δ[Ca^2+^]*_i_* and Δ[Ca^2+^]_total_ represents the Ca^2+^ buffering regardless of differential bound myofilament Ca^2+^ in control and mutant cells at baseline. Plots of [Ca^2+^]*_i_ versus* [Ca^2+^]_total_ were fitted to a Michaelis–Menten equation to give estimates of buffering *K_d_* and *B*_max_. NCX amplitudes were taken from currents obtained during the buffering measurement protocol. Relative changes in SR load were calculated using [Ca^2+^]_total_ transients upon caffeine spritz to account for the Ca^2+^ buffering of each cell. SERCA2 activity was determined from the decay constants from the caffeine transient and the preceding Ca^2+^ transient using standard methods (49).

### Measurement of sarcomere shortening and cytoplasmic Ca^2+^ transients

fura2-loaded guinea pig left ventricular cardiomyocytes were then allowed to settle to the bottom of a perfusion chamber with a 0 thickness coverslip base, which was mounted on an inverted fluorescence microscope. The cells were perfused with buffer containing 150 mm NaCl, 10 mm HEPES, 7 mm glucose, 1 mm MgCl, 1 mm KCl, 0.3 mm NaH_2_PO_3_, and 1.8 mm CaCl_2_, pH 7.4, and electrically paced at 40 V. Pacing frequency was set at 1 Hz for cells not loaded with Ca^2+^ indicator or 0.5 Hz for fura2-loaded cells to accurately measure resting diastolic [Ca^2+^]*_i_*. The effects of HCM mutations on contractility were found to be qualitatively unaltered compared with their effect in cells not loaded with fura2 at 1 Hz (Fig. S7 and Table S3). Sarcomere shortening was captured by Fourier transform of the cardiomyocyte striations under phase contrast microscopy using a switching rate of 100 Hz. Ca^2+^ transients were captured simultaneously, using the ratio of fura2 fluorescence emission at 365/380 nm at a switching rate of 1000 Hz. All contracting cardiomyocytes were measured for contractility and fura2 Ca^2+^; any cells displaying asynchronous contractility or excessive blebbing/dysmorphology were ignored for acquisition. fura2 fluorescence ratio was subsequently calibrated to [Ca^2+^]*_i_* by selective membrane permeabilization using ionomycin (Fig S1).

### Measurement of RyR leak

RyR leak was calculated using the RyR channel blocker tetracaine as previously reported (25). Briefly, contracting cardiomyocytes were identified under field stimulation at 0.5 Hz in the presence of 1.8 mm CaCl_2_. RyR leak was measured for 50 s in the absence of field stimulation in a Na^+^- and Ca^2+^-free solution. Field stimulation and perfusion of 1.8 mm CaCl_2_ were restarted to allow verification that basal functional conditions were as at the start of the experiment. Perfusion was again switched to Na^+^- and Ca^2+^-free solution containing 1 μm of tetracaine, and field stimulation was stopped to make a baseline reading that was subtracted from the preceding RyR leak measurement. Leak/load relationships were measured by 10 mm caffeine spritz following each perfusion switch.

### Western blotting and immunolocalization

Western blots were performed to detect cTnT, cTnI α-TM and FLAG tag, PLN, phosphoserine 16 PLN, phosphothreonine 17 PLN, RyR, phosphoserine 2030 RyR, phosphoserine 2808 RyR, phosphoserine 2814 RyR, phosphoserine 22/24 cTnI, NCX, SERCA2A, NFAT, phosphoserine 165 NFAT, ERK, or phosphothreonine 202/tyrosine 204 ERK in WT and mutant-infected cardiomyocytes. In immunolocalization experiments, recombinant FLAG-tagged protein was detected *versus* α-actinin. NFAT and ERK nuclear localization was also measured *versus* nucleoplasmic marker 4′,6′-diamino-2-phenylindole.

### Statistics

The data are expressed as the average of *n* experiments ± S.E. throughout. Statistically significance was determined using unpaired Student–Newman–Keuls analysis for nonnormally distributed data (InStat, GraphPad Software), with significance values defined as *p* < 0.05.

## Author contributions

P. R., Y.-H. Z., B. C., H. W., and C. R. conceptualization; P. R., X. L., A. S., S. P., and C. R. data curation; P. R., X. L., and S. P. formal analysis; P. R., B. C., H. W., and C. R. supervision; P. R., X. L., A. S., S. P., and Y.-H. Z. investigation; P. R. and X. L. methodology; P. R. and C. R. writing-original draft; P. R. project administration; P. R., X. L., Y.-H. Z., B. C., H. W., and C. R. writing-review and editing.

## Supplementary Material

Supporting Information

## Footnotes

4 The abbreviations used are:

HCM: hypertrophic cardiomyopathy
α-TM: α-tropomyosin
[Ca^2+^]_total_: total intracellular calcium
CaMKII: Ca^2+^/calmodulin-dependent protein kinase
cTnI: cardiac troponin I
cTnT: cardiac troponin T
ERK: extracellular signal-regulated kinase
MOI: multiplicity of infection
MyBPC: myosin-binding protein C
MyHC: myosin heavy chain
NCX: sodium/calcium exchanger
NFAT: nuclear factor of activated T-cells
PLN: phospholamban
RyR: ryanodine receptor
SERCA2: sarcoendoplasmic reticulum calcium ATPase 2
SR: sarcoplasmic reticulum.
