# Supplementary material for: Hypertrophic cardiomyopathy mutations increase myofilament Ca2+ buffering, alter intracellular Ca2+ handling, and stimulate Ca2+-dependent signaling
Source: J Biol Chem. 2018 May 14;293(27):10487–99. doi: 10.1074/jbc.RA118.002081 (PMC6036197; doi:10.1074/jbc.RA118.002081)
Supplement: Supporting Information [file supp_293_27_10487__index.html]

Hypertrophic cardiomyopathy mutations increase myofilament Ca2+ buffering, alter intracellular Ca2+ handling and stimulate Ca2+ dependent signalling — HCM mutations increase Ca2+ buffering and signalling — Hypertrophic cardiomyopathy mutations increase myofilament Ca2+ buffering, alter intracellular Ca2+ handling, and stimulate Ca2+-dependent signaling — HCM mutations increase Ca2+ buffering and signaling — Supporting Information 

# Hypertrophic cardiomyopathy mutations increase myofilament Ca2+ buffering, alter intracellular Ca2+ handling, and stimulate Ca2+-dependent signaling

## Supporting Information

- Supporting Information - Additional methods figures and tables
