# Supplementary material for: Hypertrophic cardiomyopathy mutations increase myofilament Ca2+ buffering, alter intracellular Ca2+ handling, and stimulate Ca2+-dependent signaling
Source: J Biol Chem. 2018 May 14;293(27):10487–99. doi: 10.1074/jbc.RA118.002081 (PMC6036197; doi:10.1074/jbc.RA118.002081)
Supplement: Supporting Information [file supp_RA118.002081_135532_2_supp_133838_p8gxk6.pdf]

Supporting Information For:

Hypertrophic cardiomyopathy mutations increase myofilament Ca^{2+} buffering, alter intracellular Ca^{2+} handling and stimulates Ca^{2+} dependent signalling

Paul Robinson*[1], Xing Liu*, Alexander Sparrow, Suketu Patel, Yin-Hua Zhang#, Barbara Casadei, Hugh Watkins, Charles Redwood

Cardiovascular Medicine Division, Radcliffe Department of Medicine, University of Oxford, UK;

* These authors contributed equally.

\# current affiliations: *Department of Physiology & Biomedical Sciences, Ischemic/hypoxic Disease Institute, Seoul National University, College of Medicine, Seoul, Korea; Yanbian University Hospital, Yanji, Jilin Province, China; Institute of Cardiovascular Sciences, University of Manchester; Manchester, UK.*

[1] To whom correspondence should be addressed: Dr Paul Robinson, Cardiovascular Medicine Division, Level 6 West Wing, John Radcliffe Hospital, Headley Way, Headington, Oxford, UK; paulr@well.ox.ac.uk; Tel.: +44 1865 234646; Fax: +44 1865 234681.

Contents:

- **Supplementary Methods**
- **Figure S1.** The calibration of fura2 fluorescence ratio $_{(F365/380)}$ to $[Ca^{2+}]_i$ in guinea pig left ventricular cardiomyocytes.
- **Figure S2.** Localisation of adenovirally expressed FLAG tagged protein in guinea pig left ventricular cardiomyocytes.
- **Figure S3.** T-tubule staining of guinea pig left ventricular cardiomyocytes following culture.
- **Figure S4.** Unloaded sarcomere shortening and Ca^{2+} transient measurements in uninfected control guinea pig left ventricular cardiomyocytes during culture.
- **Figure S5.** Titration of fura2 concentration shows that 1 mM is the optimum concentration needed for Ca^{2+} transients in adult guinea pig left ventricular cardiomyocytes after 48 hours of culture.
- **Figure S6.** Cardiomyocyte buffering protocol.
- **Figure S7.** Unloaded Sarcomere shortening of fura2 loaded cardiomyocytes.
- **Figure S8.** L-type Ca^{2+} current is not significantly altered by the presence of HCM causing mutations.
- **Figure S9.** Absolute levels of Ca^{2+} handling and myofilament proteins are unchanged by HCM mutant protein expression and pacing.
- **Figure S10.** The breakdown of $\Delta[Ca^{2+}]_i$ used to calculate RyR leak.
- **Table S1.** Average extracted parameters from sarcomere shortening and Ca^{2+} transients of fura2 loaded cardiomyocytes during culture.
- **Table S2.** Average extracted parameters from Ca^{2+} buffering measurements.
- **Table S3.** Average extracted parameters from Ca^{2+} transient and sarcomere length measurements.
- **Table S4.** Average extracted parameters from L-type Ca^{2+} and NCX current measurements acquired during Ca^{2+} buffering experiments.
- **Table S5.** Average extracted parameters from $[Ca^{2+}]_i$ and $[Ca^{2+}]_{total}$ SR Load and Ca^{2+} transients following 5 second pause.
- **Table S6.** Average extracted parameters from $[Ca^{2+}]_i$ SR Load measurements acquired without pause.
- **Table S7.** Average extracted parameters from RyR leak experiments.
- **Table S8.** Densitometry and image analysis comparing NFAT and ERK phosphorylation levels and NFAT and ERK nuclear localisation in paced vs unpaced cardiomyocytes comparing WT and mutant infected cardiomyocytes.

Supplemental Material

Expanded Methods:

Virus design and production

Recombinant adenoviral constructs were created using the AdEasy recombination system (Agilent technologies) as previously described.(1) Briefly; WT cTnT, R92Q cTnT, WT cTnI and R145G cTnI containing an N-terminal -DYKDDDDK- FLAG Tag and WT α-TM and D175N α-TM containing a C-terminal -DYKDDDDK- FLAG Tag, were cloned in to a shuttle vector, containing a CVM promoter 3' of the multiple cloning site and an inter-ribosomal expression site linked to a hrGFP gene at the 5' end. The shuttle vectors were recombined in BJ5183-AD1 *E.coli* that had been pre-transformed with the AdEasy viral backbone plasmid. Colonies positive for homologous recombination were confirmed with PacI and BstxI digest patterns. Successfully cloned AdEasy plasmids were linearised and transfected into an 80 % confluent 25 cm^2 tissue culture flask containing genetically modified (AD) HEK 293 cells that package the adenoviral particles for 3-4 weeks. Scale up of high titre adenovirus was achieved by sequential rounds of re-infection from cell lysates using increasing cell numbers to a final infection of fifty 175 cm^2 flasks of 90 % confluent AD293 cells. The viral particles from this round of infection were purified using overnight CsCl gradient centrifugation and desalted in to a buffer containing 20 mM tris-HCl pH 7.0 and 7.5% sucrose. Desalted virus was mixed 1:1 with a solution containing 20 % glycerol 80% FBS for cryo- protection and stored at -80 °C. Viral titre estimation was carried out estimating the number of green cells infected by serial dilutions of purified virus in a plate containing ~$2x10^6$ unmodified HEK293 cells over a 48 hour period. Estimates showed that the number of viable virus particles was between $1x10^{10}$ and $7x10^{11}$.

Isolation of guinea pig left ventricular cardiomyocytes

A 400 g male guinea pig was dispatched by cervical dislocation. The chest cavity opened, an 18 gauge cannula inserted into the aorta and fixed in place with a short length of sterile suture. The heart was excised and immediately hung on langendorff apparatus which was water jacketed to 37 °C and in

which all solutions were preoxygenated. The heart was perfused with isolation solution (130 mM NaCl, 23 mM 4-(2-hydroxyethyl)-1-piperazineethanesulphonic acid (HEPES), 21 mM glucose, 20 mM taurine, 5 mM creatine, 5 mM MgCl , 5 mM Na pyruvate, 4.5 mM KCl, 1 mM NaH_2PO_3, pH 7.3 with NaOH) containing 0.016 mM EGTA for 5 minutes. The heart was subsequently perfused with isolation solution containing 0.15 mM $CaCl_2$, 0.1 % BSA, 0.8 mg/ml collagenase type II (Worthington) and 0.06 mg/ml of protease from streptomyces griseus (sigma) for 5 minutes, followed by perfusion with isolation solution containing 0.15 mM $CaCl_2$, 0.1% BSA for 5 minutes to inactivate the collagenase. The left ventricle was cut away, mechanically homogenised and placed in 50 ml of isolation solution containing 0.15 mM $CaCl_2$, 0.1 % BSA in a shaker at 37 °C for 10-15 minutes until a significant proportion of the excised tissue had broken up. Isolated cells were passed through a sterile gauze filter to remove any undigested tissue and centrifuged for 2 minutes at >100 xg, the supernatant was removed and the cell pellet was resuspended 50 ml isolation solution containing 0.5 mM $CaCl_2$ and immediately centrifuged for 2 minutes at >100 xg , the supernatant was removed, the cell pellet was resuspended in 50 ml of isolation solution containing 1 mM $CaCl_2$ and immediately centrifuged for 2 minutes at >100 xg and the supernatant was removed. Finally the cells were resuspended and plated at ~$1x10^5$ per ml in $ACCITT_3$ cardiomyocyte culture medium first described by Ellingsen et al(2,3) containing 500 ml M199 media 100 units of penicillin, 0.1 mg/ml streptomycin, 20 mM taurine, 5 mM creatine, 5 mM Na pyruvate, 2 mg/ml BSA, 2 mM L-carnitine, 0.1 PM insulin. Recombinant adenovirus was immediately added to 3 ml of cell suspension to an estimated multiplicity of infection (MOI) of ~1000, infected cells were placed at 37 °C in a 5 % CO_2 atmosphere for 48 hours.

Measurement of cardiomyocyte t-tubules using di-4-ANEPPS

Isolated cardiomyocytes were cultured in $ACCITT_3$ media for 0, 24 or 48 hours, 1 μM of di-4-ANEPPS and 2 μM Pluronic® F-127 (Life Technologies) was added directly of the culture media for 20 minutes, Cells were washed with fresh media, and returned to a fresh 35 mM tissue culture dish. They could then be visualised immediately using a Leica TCS SP5 X confocal microscope equipped

with a 433 nm argon laser and a 20X aqueous dipping lens. Images were acquired using a photomultiplier set between 500-600 nm for optimum image resolution and fluorescence intensity. Di-4-ANEPPS specifically co-ordinates with voltage gated ion channels on the t-tubule membrane of the cardiomyocyte. The extent of t-tubulation was estimated by pixel counts of 25 images acquired by z-stack per cell following export to image J analysis software.

The Calibration of $[Ca^{2+}]_i$

The ratio of fura2 fluorescence signal measured at 365 and 380 nm was converted to $[Ca^{2+}]_i$ using a Ca^{2+} calibration kit with Mg^{2+} (Life Technologies). Isolated cardiomyocytes were loaded with 1 μM of fura2 as previously described. Next the cells were incubated in a perfusion solution containing 5 μM thapsigargin (sigma) and 10 mM caffeine for 20 minutes, to ensure complete unloading of SR Ca^{2+}. Finally, cells were divided into aliquots and incubated with 10 μM Ionomycin (Life Technologies) to specifically permeablize the cell membrane to Ca^{2+} and 30 mM 2,3-butanedione (BDM) to inhibit myocyte contraction. Each aliquot of cells was exposed to one in a range of 10 mM K_2-EGTA/Ca-EGTA buffers containing 0, 4.0, 8.0, 8.25, and 8.5 mM CaEGTA (equivalent to free $[Ca^{2+}]$ of 0, 0.1, 0.6, 2.4, and 3.9 μM) The buffers also contained 100 mM KCl, 1 mM $MgCl_2$ and 30 mM MOPS, pH 7.2. Fluorescence ratio was plotted against free $[Ca^{2+}]$ (Fig S1). The resultant exponential equation was used in all subsequent measurements to covert the observed $F_{365/380}$ to $[Ca^{2+}]_i$.

Measurement of Ca^{2+} Buffering, L-type Ca^{2+} current, SR Ca^{2+}, NCX and SERCA2a activity

Ca^{2+} buffering was measured in accordance with the protocol established by Trafford, *et al(4)*. Briefly, Infected guinea pig LV myocytes were loaded with fura-2AM (1 μM) as described above and then mounted onto the electrophysiology set-up with Normal Perfusion Solution containing 1.8 mM $CaCl_2$ at 37°C. The cell was then patched with a pipette containing (in mM): K-glutamate 120, KCl 30, HEPES 10, $MgCl_2$ 5, KATP 5, Na_2-creatine phosphate 3.6, NaCl 2.8, cAMP 0.05; pH = 7.2 (adjusted with KOH). Whole cell voltage-clamp and current clamp experiments were carried out at 37°C using

an Axopatch 200B amplifier and Digitata 1322A data-acquisitioμn system (Axon Instruments). The electrode resistance is ranged from 2-4 MΩ. After rupture of the membrane, membrane capacitance (C_m) was routinely measured and calculated from responses to 10 mV hyperpolarizing and depolarizing step changes in membrane voltage (ΔV_m) applied from a holding potential of -70 mV. Every 1 second (1 Hz) the cell was depolarized to 0 mV for 100 ms from holding potential at -40 mV to elicit a $[Ca^{2+}]_i$ transient until it achieved a steady-state waveform that was characterized by reproducible resting and peak levels of $[Ca^{2+}]_i$. The peak current, fast and slow decay rates and integral were taken from these initial currents to give a measure of L-Type Ca^{2+} channel activity. Caffeine (10 mM) was then applied for 10 seconds following a 5 second pause. The resulting $[Ca^{2+}]_i$ transient and the concurrent inward, electrogenic current via the Na^+/Ca^{2+} exchanger (NCX) upon applying the caffeine were simultaneously recorded (Figure 5A). Steady-state $[Ca^{2+}]_i$ transient stimulation was then resumed.

The time-course of change of total Ca^{2+} in the cell was obtained by integrating the NCX current backward in time during Caffeine application. The integral was then corrected for non NCX exchange fluxes(4). This was compared with the measurement of $[Ca^{2+}]_i$ during the same period as means of free intracellular Ca^{2+} to obtain a buffering curve. This curve could be fitted according to the following formula, which gives a value for the maximum Ca^{2+} buffering capacity of the intrinsic buffers (B_{max}) as well as K_d:

$$\text{Total } Ca^{2+} = \{B_{max} * [Ca^{2+}]_i / (K_d + [Ca^{2+}]_i)\} + B_{min}$$

Relative buffering capacity in low (>1 μM) Ca^{2+} can to be calculated by K_d / B_{max}.

The relative SR Ca^{2+} load, fractional release Ca^{2+} release and SERCA activity was calculated for both $[Ca^{2+}]_i$ and $[Ca^{2+}]_{total}$ for each individual cell measured during the buffering experiments. SR load was assumed to be the Caffeine induce $[Ca^{2+}]_{total}$ transient amplitude, Fractional Ca^{2+} release from the SR was calculated by the division of SR $[Ca^{2+}]_{total}$ (given by the caffeine transient amplitude) by the $[Ca^{2+}]_{total}$ transient amplitude preceding caffeine application. NCX peak current was also derived from voltage clamp recordings during the buffering measurements by observing the peak NCX current given during 10 mM caffeine application. SEACA2 activity was calculated by the subtraction of the

fast $[Ca^{2+}]_{total}$ transient τ_1 decay rate (which gives the total intracellular Ca^{2+} reuptake) from the slower τ_2 decay of the $[Ca^{2+}]_{total}$ caffeine transient (which gives the NCX activity).

SR Load was independently calculated in a separate experiment where 10 mM caffeine (in Ca^{2+} perfusion buffer) was applied immediately after deactivation of pacing using a separate gravity perfusion system attached to a heated (37 °C) perfusion pencil (Digitimer). Electrical field stimulation was restarted after 20 seconds of constant caffeine application. Switching of pacing and caffeine application were automated using Clampex software (Axon Instruments) to ensure it was instantaneous.

We examined whether there is any alteration in L-Type Ca^{2+} current and total Ca^{2+} pump inside the cell upon action potential as Ca^{2+} current is the initiator of Ca^{2+}- induced Ca^{2+} release. Our recording shows that both peak L-Type Ca^{2+} current and total Ca^{2+} integral are unchanged between all three HCM mutants and their matched WTs. The decay of the Ca^{2+} current fitted using double-exponential function. The fast component of decay was not significantly different, whereas the slow time constant was significantly greater in R92Q myocytes than in matched wild type control only.

Measurement of sarcomere shortening and Ca^{2+} transients

Sarcomere shortening and Ca^{2+} transient measurements were performed using IonOptix μstep apparatus and the manufacturers' standard operating instructions. Briefly: cultured cardiomyocytes were loaded with fura2 Ca^{2+} indicator by incubation with 1 μ fura2-AM ester (Life Technologies) in the presence of 2 μM F127 pluronic in 'perfusion buffer' (150 mM NaCl, 10 mM 4-(2-hydroxyethyl)-1-piperazineethanesulphonic acid (HEPES), 7 mM glucose, 1 mM MgCl , 1 mM KCl, 0.3 mM NaH_2PO_3, pH 7.4 with NaOH) containing 250 μM $CaCl_2$ for 5 minutes, followed by a 10 minute wash in perfusion buffer containing 500 μM $CaCl_2$ to remove any excess label. The loaded cells were then allowed to settle to the bottom of a perfusion chamber with a 0 thickness cover slip base, which was mounted on an inverted fluorescence microscope. Cells were perfused with Perfusion buffer containing 1.8 mM $CaCl_2$, and electrically paced at 40 volts. Pacing frequency was set at 1 Hz

for cells not loaded with Ca^{2+} indicator. However, due to the buffering capacity of the dye, the fura2-loaded cardiomyocytes had significantly reduced contractile amplitude and velocity compared with unloaded cells and we therefore chose to decrease the pacing frequency to 0.5 Hz in order to accurately measure resting diastolic $[Ca^{2+}]_i$. The effects of HCM mutations on contractility were found to be qualitatively unaltered compared to their effect in cells not loaded with fura2 at 1 Hz (Fig S7 and Table S4). Sarcomere shortening was captured by fourier transform of the cardiomyocyte striations under phase contrast microscopy using a switching rate of 100 Hz. Ca^{2+} transients were captured simultaneously, using the ratio of fura2 fluorescence emission at 360/380 nm at a switching rate of 1000 Hz. All contracting cardiomyocytes were measured for contractility and fura2 Ca^{2+}, any cells displaying asynchronous contractility, excessive blebbing/dysmorphology were ignored for acquisition, whilst any cell with contractile magnitudes or velocities exceeding 2 standard deviations from the mean upon analysis were also excluded as product of phenotypic heterogeneity of primary cells in culture losing function and morphology over time.

Measurement of RyR leak

The rate of Ca^{2+} leak from the SR via the RyR can be measured using the channel blocker tetracaine, in a method adapted from Shannon et al(5). Briefly, a contracting fura2 loaded cell was identified under constant perfusion of 1.8 mM Ca^{2+} perfusion buffer and electrical field stimulation of 0.5 Hz, the perfusate was systemically switched to a solution containing no Ca^{2+} or Na^+ and electrical field stimulation was stopped for 100 seconds ending with the direct application of 10 mM caffeine to assess SR Ca^{2+} content as described above. The systemic perfusion was returned to normal Ca^{2+} perfusion solution and electric field stimulation was restarted to allow the cell to recover to pre-challenge levels of contractility and Ca^{2+} transient amplitude. After recovery, the solution was switched to a no Ca^{2+} or Na^+ solution containing 1 µM tetracaine, whilst electrical field stimulation was again stopped for 100 seconds. Finally, a second direct application of 10 mM caffeine was given to assess SR Ca^{2+} content under RyR blockade. It was also found that there were small but significant reductions to the fura2 signal and fatigue/ reduced contractility over the time course of the experiment. This was because each experimental solution was washed out with a regular perfusion

solution containing 1.8 mM $CaCl_2$ before the next was applied. In order to prevent experimental bias an average of 30 cells were measured in each group; at least 15 cells were acquired as shown in Figure 6A whilst a further 15 were acquired by reversing the sequence of Na^+ Ca^{2+} free solutions with or without 1 μM tetracaine.

Western blotting

Western Blots samples were prepared from 6x10^5 infected cardiomyocytes precisely 48 hours after viral infection. To assess recombinant protein expression levels, cardiomyocytes we imaged under both phase contrast and FITC fluorescent microscopy to accurately determine the percentage infection levels for each sample of each virus. Samples were blotted using either mouse anti-FLAG-Tag (1/1000) (Sigma), mouse anti-cTnT (JLT clone) (1:1000) (Sigma), mouse anti-cTnI (1:2000) (Millipore), mouse anti-α-TM (1:2000) (Sigma) over night at 4 °C, followed with a horseradish peroxidase conjugated bovine anti-mouse IgG secondary antibody (1:4000) at room temperature for 1 hour. All membranes were stripped by incubation with Restore Plus reagent (Thermo Fisher) for 10 minutes at room temperature and re probed with anti-GAPDH (1:2000) (Millipore) primary and a bovine anti rabbit HRP secondary (1:8000) for loading control assessment. The incorporation of recombinant protein was calculated using the relative band intensities of endogenous vs FLAG tagged recombinant protein for each infection, adjusted by the previously acquired infection levels for each sample prepared. Relative recombinant protein levels were assessed in this way every 2-3 months throughout the experimental time course to check for viral titre rundown due to cryostorage.
Samples for Ca^{2+} handling and signalling were prepared using 3x10^5 cells, plated in 35 mm dishes and infected with the appropriate MOI of virus for 48 hours. Cells were either paced at 0.5 hz for between 4 and 8 hours (using IonOptix C-pace electrode array) or left unstimulated; they were then resuspended in 200 μl of CellLytic MT cell lysis buffer (Sigma) containing both protease and phosphatase inhibitors. Cells were homogenised using a Sonoplus probe sonicator (Bandelin) pulsing at 10 % power for 30 seconds followed by vigorous mechanical agitation using a 25 gauge syringe. SDS-PAGE loading buffer (50 μl) was added without heat denaturation to preserve visualisation of both the penta and monomeric PLN. All other samples for TnI, NCX, SERCA, NFA and ERK were

heated to 95 °C for 5 minutes before loading on 4-12 % gradient Gels for blotting. Samples for RyR western blots were prepared in the same manner until lysis was completed at which time each sample was incubated with 50 mg/ml anti-RyR antibody (Millipore) overnight at 4 °C. RyR was immunoprecipitated using 50 µl of protein A/G plus agarose (Santa Cruz) following the suppliers wash protocol. Purified RyR was finally eluted in 50 µl of CellLytic MT cell lysis buffer (Sigma), 15 µl of SDS-PAGE loading buffer was added prior to heat denaturation at 95 °C for 10 minutes. Samples for RyR were run on 3-8 % Tris Acetate gradient gels (Life Technologies). Membranes were incubated overnight at 4 °C with either anti-PLN (1:4000) (Badrilla), anti-phospho-serine 16 PLN (1:4000) (Millipore), anti-phospho-threonine 17 PLN (1: 2000) (Santa Cruz), anti-RyR (1:1000) (Millipore), anti-phospho-serine 2030 RyR (1:1000) (Badrilla), anti-phospho-serine 2808 RyR (1:2000) (Badrilla) or anti-phospho-serine 2814 RyR (1:2000) (Badrilla), anti-cTnI (1:2000) (Aviva Systems Biology), anti-phospho-serine 22/24 cTnI (1:1000) (Cell Signalling Technologies), anti-NCX (1:2000) (Santa Cruz), anti SERCA2A (1:5000) (Santa Cruz), Anti NFAT (1/2000) (Santa Cruz), anti-phospho-serine -165 NFAT (1 in 3000) (Invitrogen), anti ERK (1/3000) (Cell signalling) or anti-phospho-threonine 202/ tyrosine 204-ERK (1/2000) (Cell Signalling). This was followed five washes in PBST for 5 minutes and then incubated with a horseradish peroxidase conjugated bovine anti-mouse IgG (Santa Cruz) or goat anti-rabbit IgG (Promega) secondary antibody (1:4000) at room temperature for 1 hour. Bands were visualised by exposing the membranes with ECL select substrate (GE healthcare) for 5 minutes. Images were acquired using a gel documentation imaging system (Bio Rad). Antibodies were stripped using 10 ml of Restore™ western blot stripping solution (Thermo Scientific) at room temperature for 8-10 minutes. Membranes were reprobed with an anti-α-actinin (1:4000) (Sigma) that served as a loading control. The intensity of the bands was determined using Image J densitometry analysis (NIH).

Immunolocalisation

For the detection of the localisation of human recombinant FLAG-Tagged cTnT, cTnI and α-TM; 2-well chamber slides (BD Bioscience) were pre-coated with 40 µg/ml laminin (BD Bioscience) in PBS at room temperature for 3 hours. They were then washed thoroughly with PBS and approximately

1x10^5 freshly isolated and infected cardiomyocytes were plated in ACCITT$_3$ medium. After incubation at 37 °C in a 5 % CO_2 atmosphere for 48 hours the medium was removed, adherent cells were washed in PBS, and immersed in a 0.05 % Triton-X100 PBS solution at room temperature, in order to chemically skin the cells. The skinning process was followed for ~10-15 minutes by observing GFP leak from the cells using a FITC fluorescence microscope. The skinning reaction was stopped by the rapid removal of the Triton-X100 solution and three washes in PBS. Cells were fixed for antibody staining with the application of 4 % methanol free paraformaldehyde (TAAB) for 15 minutes followed by two washes with PBS. Finally, the cells were blocked with a 5 % BSA solution for 30 minutes. Primary antibodies: mouse anti-FLAG-tag (1:8) (Sigma) and rabbit anti-α-actinin (Sigma) (1:500) were incubated overnight at 4 °C. Secondary antibodies: goat anti mouse IgG Alexa 564 (1:200) (Life Technologies) and goat anti-rabbit IgG Alexa 633 (1:200) (Life Technologies) were incubated at room temperature in darkness for 2 hours. The gasket surrounding the chamber slide was removed and mounted with SlowFade gold anti-fade reagent with DAPI (Life Technologies) was applied and a square 0 thickness 24 mm coverslip was fixed in place over the cells. Slides were then visualised using a Leica TCS SP5 X confocal microscope with a 63x oil immersion objective.

For detection of the nuclear localisation of NFAT-c3 and ERK, round coverslip bottomed 35 mm culture dishes (Matek) were coated with laminin and plated with infected cardiomyocytes as above. After 48 hours incubation at 37 °C in 5 % CO_2, the media was removed and adherent cells were fixed for 15 minutes with 4 % methanol free paraformaldehyde. They were then blocked and skinned in 5 % BSA and 0.05 % Triton-X100 solution for 30 minutes at room temperature. Primary mouse: anti-NFAT-c3 (1/50) (Santa Cruz) or Anti-ERK (1 in 100) (Cell Signalling) was incubated over night at 4 °C. Secondary antibody: goat anti-mouse IgG Alexa 568 (1/200) (Life Technologies) was incubated at room temperature in darkness for 2 hours. Slow fade slide mount with DAPI (Life Technologies) was applied and a circular 0 thickness 19 mm round coverslip was fixed in place over the cells. The sides of the 35 mm culture dish were removed using a heated scalpel blade, the cover slip containing the cells was glued to a microscope slide and visualised using a Leica TCS SP5 X confocal microscope as above.

Statistics

The data are expressed as the average of n experiments±SEM (SE). Statistically significant differences were determined using an unpaired Student Newman Kewls t test (InStat, GraphPad Software), with significance values defined as $P<0.05$. For all sarcomere length and Ca^{2+} transient measurements, any cell presenting with an extracted parameter greater than two standard deviations from the mean were removed from the final data set. This was to prevent cells with significant alterations of function due to damage from isolation and culture protocols from biasing the final results.

1. Luo, J., Deng, Z. L., Luo, X., Tang, N., Song, W. X., Chen, J., Sharff, K. A., Luu, H. H., Haydon, R. C., Kinzler, K. W., Vogelstein, B., and He, T. C. (2007) A protocol for rapid generation of recombinant adenoviruses using the AdEasy system. *Nature protocols* **2**, 1236-1247
2. Ellingsen, O., Davidoff, A. J., Prasad, S. K., Berger, H. J., Springhorn, J. P., Marsh, J. D., Kelly, R. A., and Smith, T. W. (1993) Adult rat ventricular myocytes cultured in defined medium: phenotype and electromechanical function. *Am J Physiol* **265**, H747-754
3. Bistola, V., Nikolopoulou, M., Derventzi, A., Kataki, A., Sfyras, N., Nikou, N., Toutouza, M., Toutouzas, P., Stefanadis, C., and Konstadoulakis, M. M. (2008) Long-term primary cultures of human adult atrial cardiac myocytes: cell viability, structural properties and BNP secretion in vitro. *International journal of cardiology* **131**, 113-122
4. Trafford, A. W., Diaz, M. E., and Eisner, D. A. (1999) A novel, rapid and reversible method to measure Ca buffering and time-course of total sarcoplasmic reticulum Ca content in cardiac ventricular myocytes. *Pflugers Arch* **437**, 501-503
5. Shannon, T. R., Ginsburg, K. S., and Bers, D. M. (2002) Quantitative assessment of the SR Ca2+ leak-load relationship. *Circ Res* **91**, 594-600

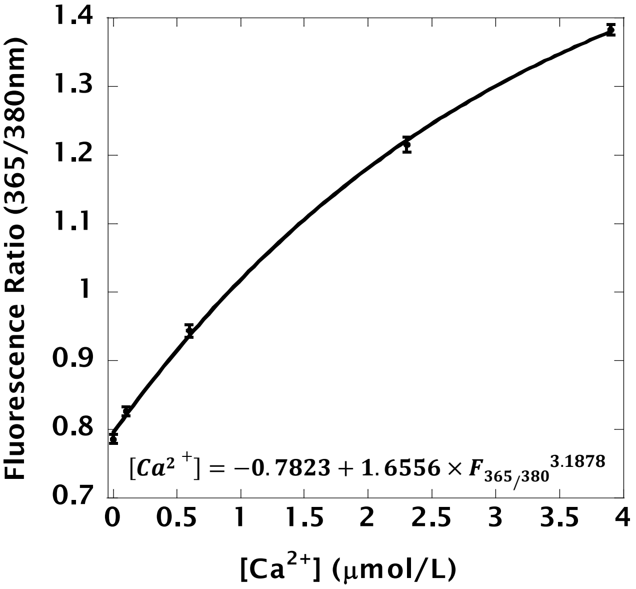

Figure S1. The calibration of fura2 fluorescence ratio ($F_{365/380}$) to $[Ca^{2+}]_i$ in guinea pig left ventricular cardiomyocytes. Fluorescence ratio measurements ($F_{365/380}$) plotted on the Y axis were taken from membrane permiablized guinea pig left ventricular cardiomyocytes loaded with 1 μM fura2. Free extracellular $[Ca^{2+}]$ plotted on the X axis was set by incubating cells in 10 mM EGTA and either 0, 4.0, 8.0, 8.25, and 8.5 mM CaEGTA (equivalent to free $[Ca^{2+}]$ of 0, 0.1, 0.6, 2.4, and 3.9 μM). The resultant curve was fitted to a single exponential equation, shown in the inset, to allow calibration of all subsequent Ca^{2+} transient measurements.

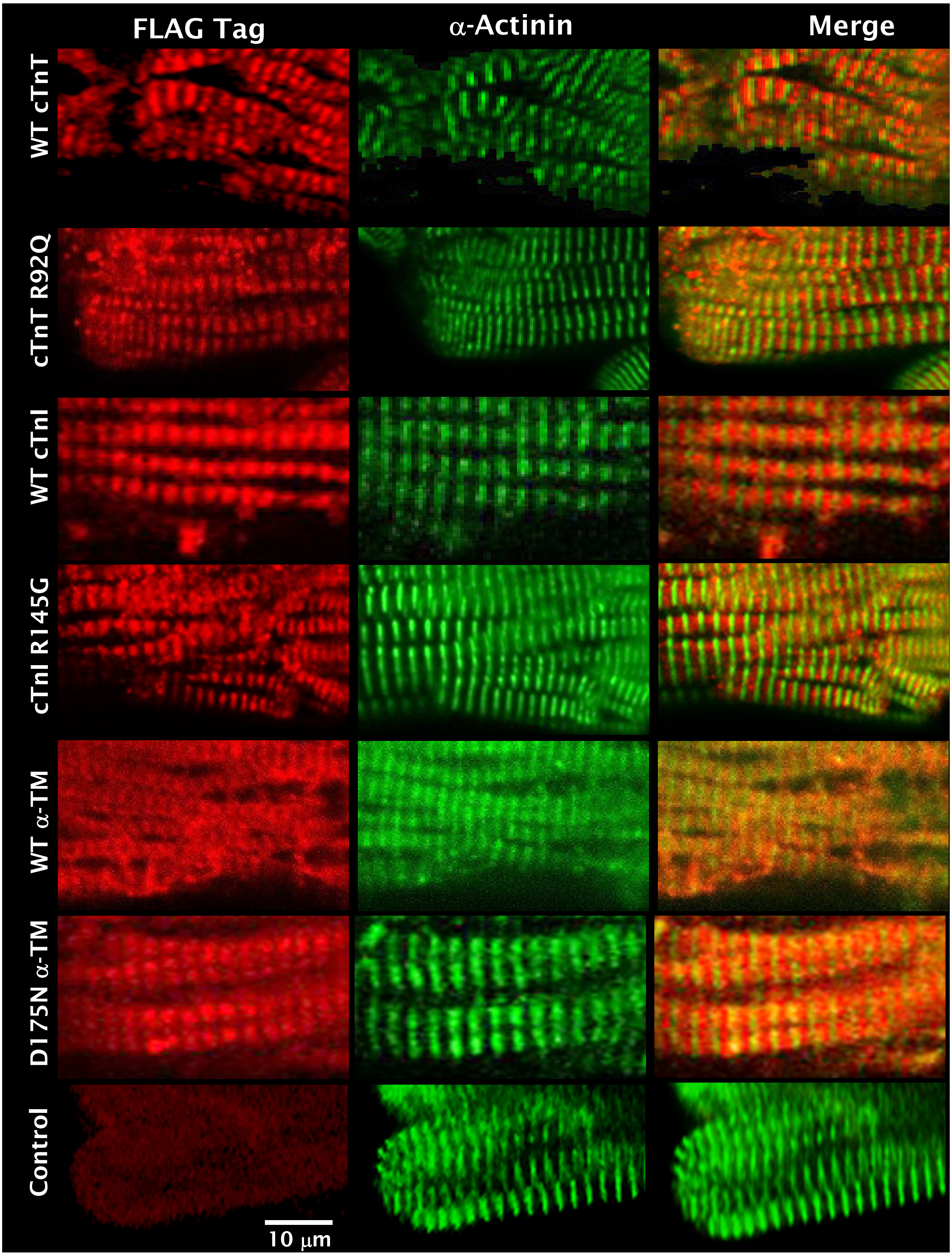

Figure S2. Localisation of adenovirally expressed FLAG tagged protein in guinea pig left ventricular cardiomyocytes. Adenovirally expressed FLAG tagged protein was localised to the I band in cardiomyocytes fixed to laminin coated cover slips using and anti-FLAG tag primary antibody (conjugated to Alexa568, red), with counterstain provided using an α-actinin antibody (conjugated to Alexa 633, false coloured green) to stain the z disks.

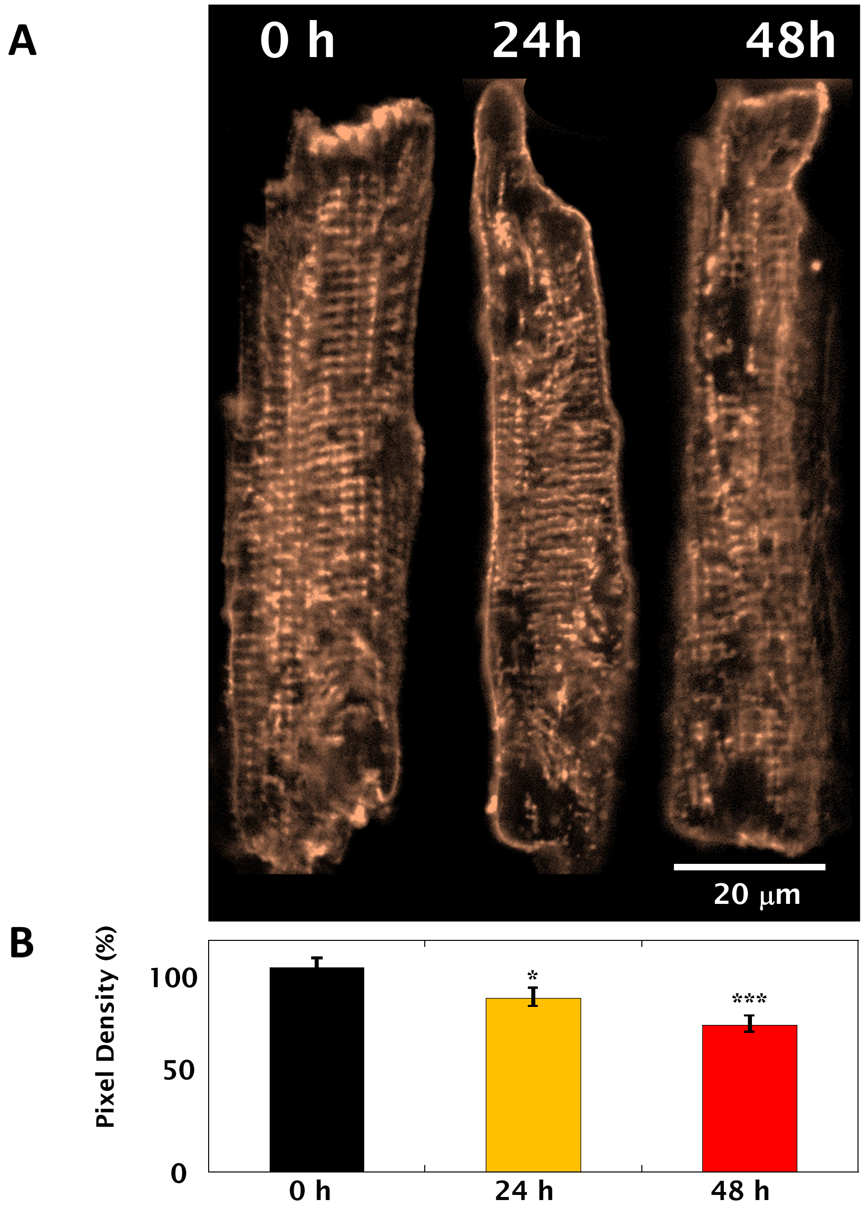

Figure S3. T-tubule staining of guinea pig left ventricular cardiomyocytes following culture. The cells in A show the extent of t-tubulation of guinea pig left ventricular cardiomyocytes after 0, 24 and 48 hours in our standard culture conditions compared to freshly isolated cells. Confocal images of t-tubules were obtained by loading the cells with 1 µM di-4-ANEPPs and imaging the live cells using a 20x dipping lens using argon laser excitation and an emission range between 550 and 700 nm. The relative percentage t-tubulation shown in B was estimated by pixel counts of a fixed 50 x 15 µm representative area positioned in the centre of the cell, 10 cells were measured in each group. Significance values shown are $p<0.001$=*** and $p<0.05$=*. Error bars are the SEM.

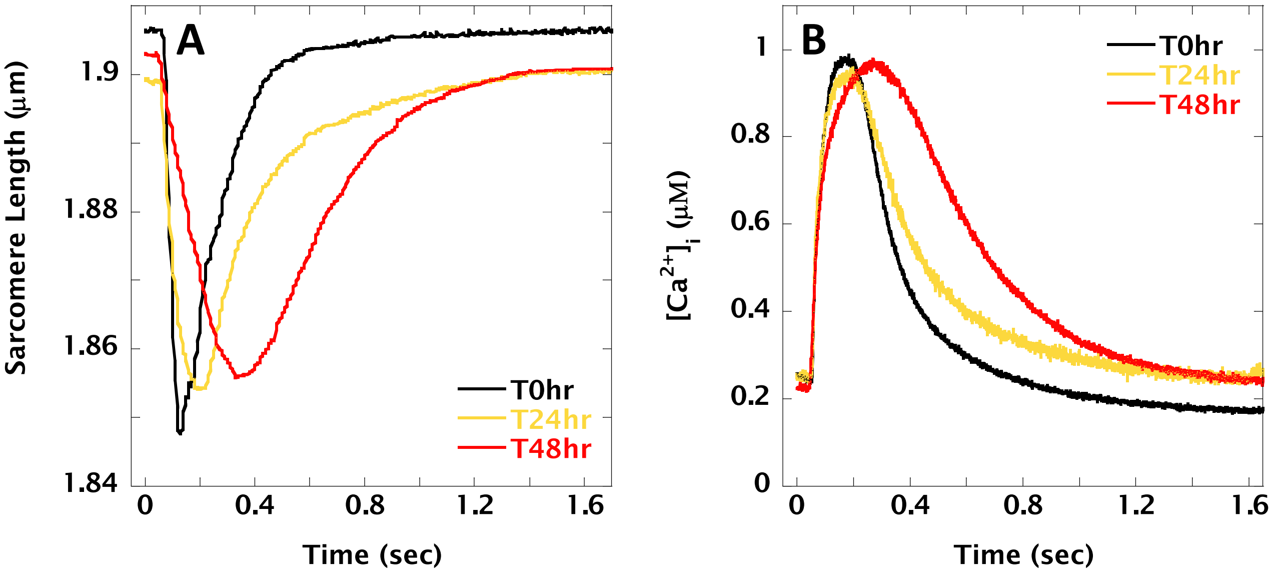

Figure S4. Unloaded sarcomere shortening and Ca^{2+} transient measurements in uninfected control guinea pig left ventricular cardiomyocytes during culture. Unloaded sarcomere shortening measurements in A, compares the effect culturing cardiomyocytes under standard conditions for 24 and 48 hours compared to freshly isolated cells at a pacing frequency of 0.5 Hz. B shows the corresponding intracellular Ca^{2+} transients of the same cardiomyocytes loaded with 1 μM fura2. Each curve was averaged from 10-15 cells taken from at least 4 separate cell preparations.

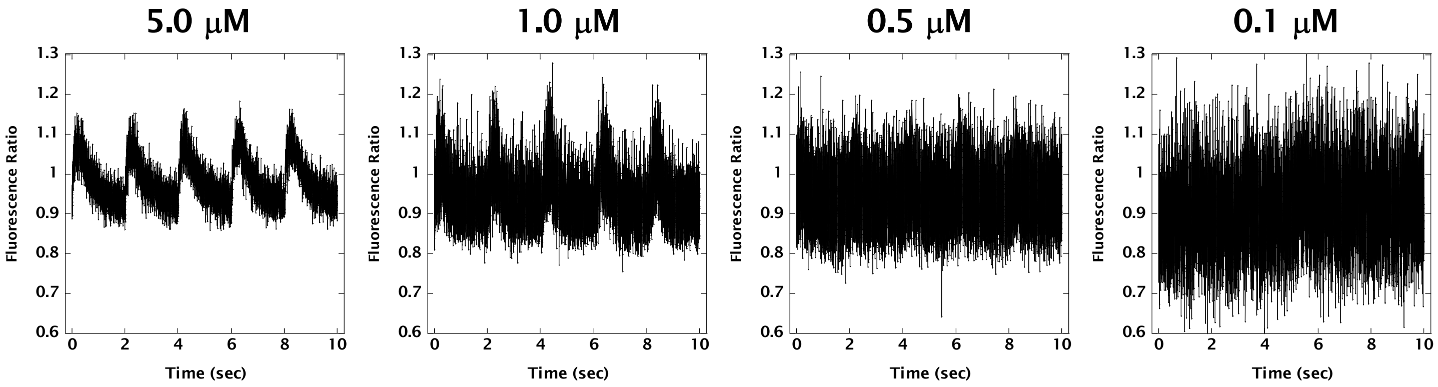

Figure S5. Titration of fura2 concentration shows that 1 μM is the optimum concentration needed for Ca^{2+} transients in adult guinea pig left ventricular cardiomyocytes after 48 hours of culture. Unloaded sarcomere shortening measurements in A, compares the effect on relative fluorescence ration $F_{340/380}$ of loading , 5, 1, 0.5 and 0.1 μM of the calcium indicator fura2 at a pacing frequency of 0.5 Hz Each curve was averaged from 15-20 cells taken from at least 3 separate cell preparations. Increasing concentrations of fura2 reduce the contractile magnitude by buffering the intracellular Ca^{2+} in our particular cell models system, however until a concentration of 1 μM is reached the fura2 fluorescence signal/ noise ratio is too low.

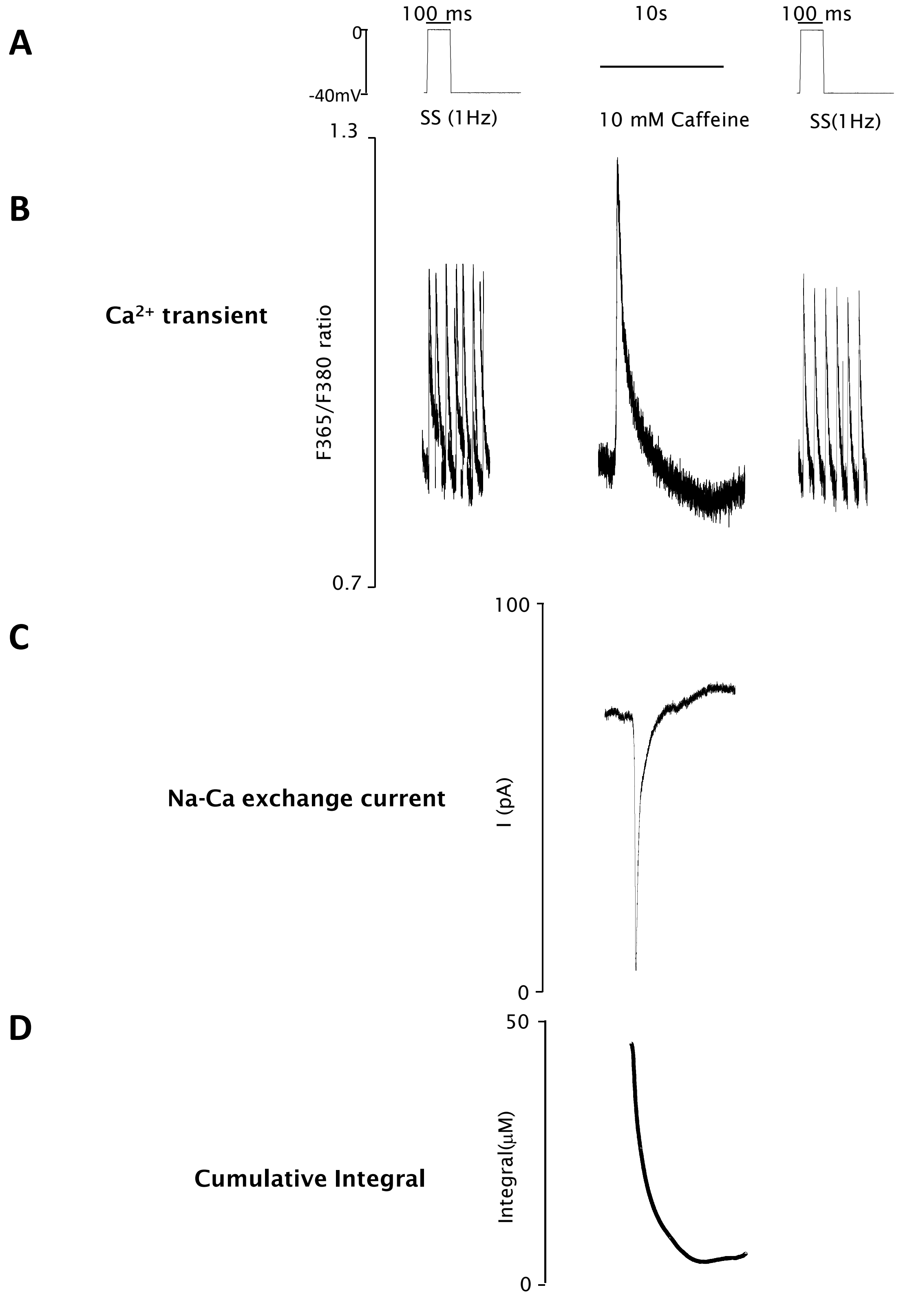

Figure S6. Cardiomyocyte buffering protocol. A shows the voltage change via the voltage clamp electrode. B shows a representative Ca^{2+} transient measured by fura2 fluorescence ratio (365/380 nm). C shows the corresponding NCX current during the application of 10 mM caffeine. D shows the "total Ca^{2+} (μM)" derived from the integral of the NCX current in C.

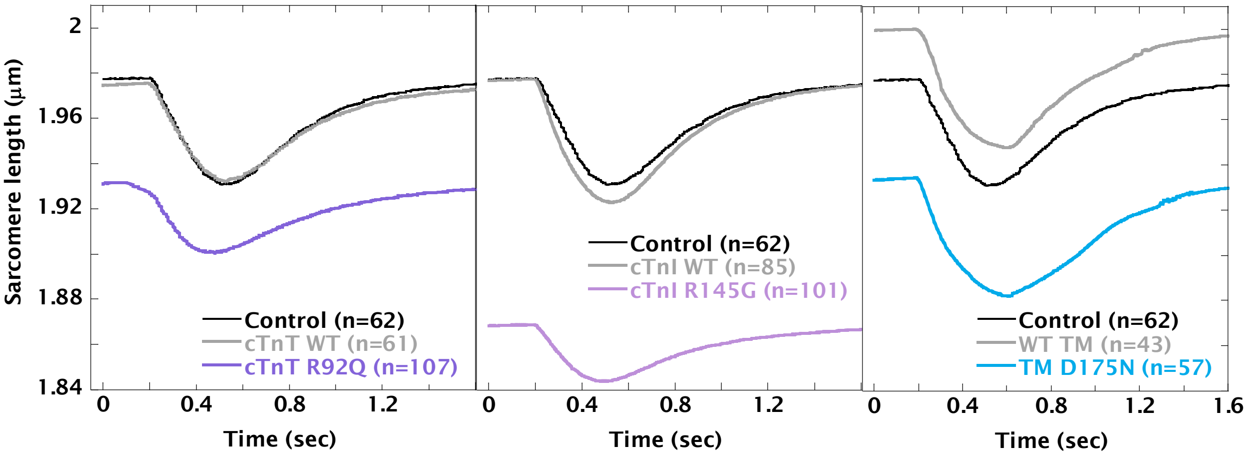

Figure S7. Unloaded Sarcomere shortening of fura2 loaded cardiomyocytes. Unloaded sarcomere shortening measurements compares the effect HCM causing mutations cTnT R92Q, cTnI R145G and α-TM D175N to cardiomyocytes infected with WT human recombinant cTnT and uninfected control cells at a pacing frequency of 0.5 Hz. Each curve was averaged from multiple cells taken from at least 4 separate cell preparations, total n numbers are given in the insert of each plot

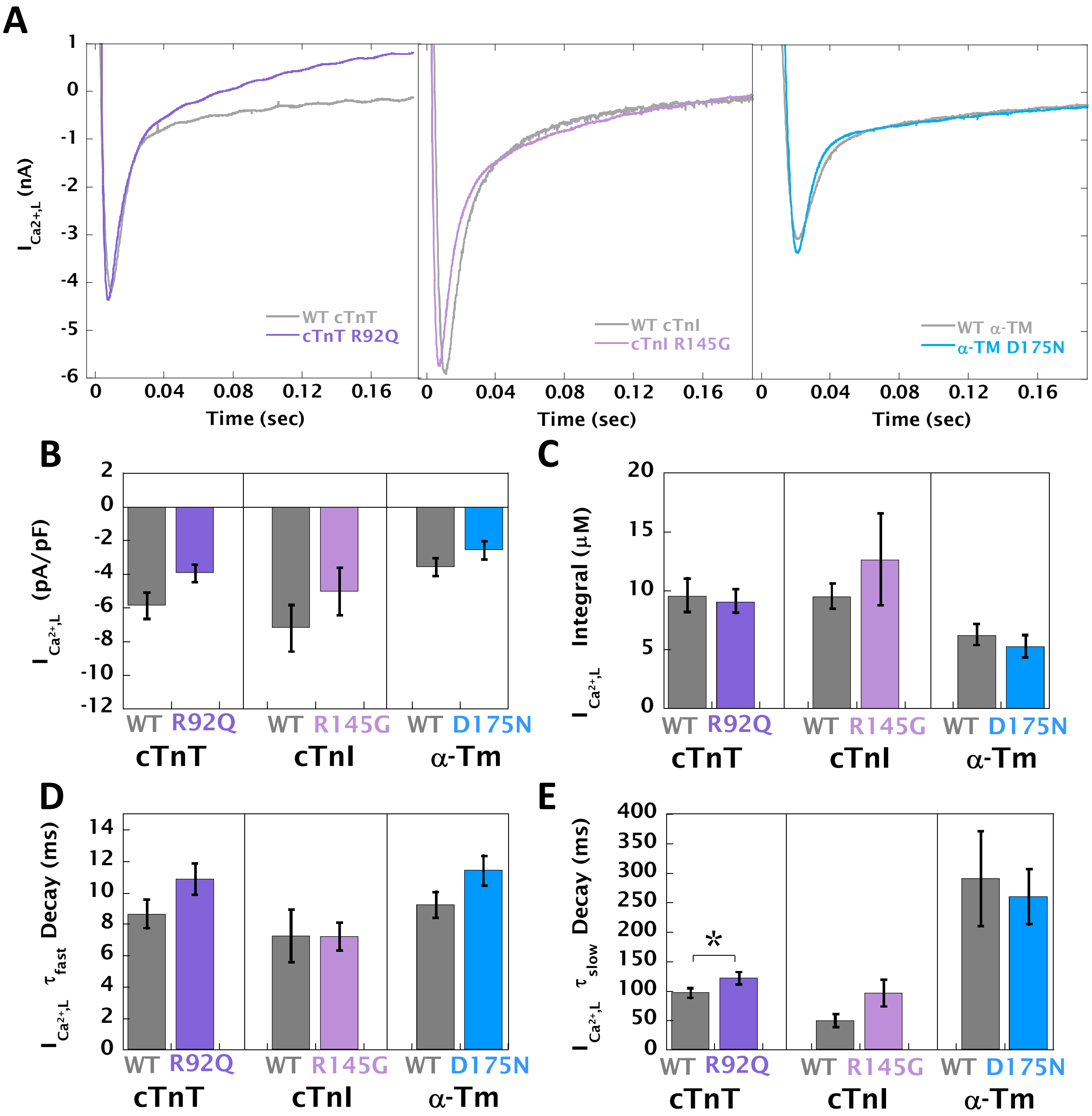

Figure S8. L-type Ca^{2+} current is not significantly altered by the presence of HCM causing mutations. A shows representative traces to illustrate L-type Ca^{2+} current ($I_{Ca2+,L}$) to compare WT (n=22-11) and HCM mutant (n=25-12) transfected cardiomyocytes using whole cell voltage clamp. Bar graphs B, C D and E show average extracted parameters Peak current ($I_{Ca2+,L\ peak}$ (pA/pF)), current integral ($I_{Ca2+,L\ integral}$ (μM), fast decay constant ($I_{Ca2+,L}\ \tau_{fast}$ (ms)), and slow decay constant ($I_{Ca2+,L}\ \tau_{slow}$ (ms)) respectively. Significance values shown are $p<0.001$=*** and $p<0.05$=*. Error bars are the SEM.

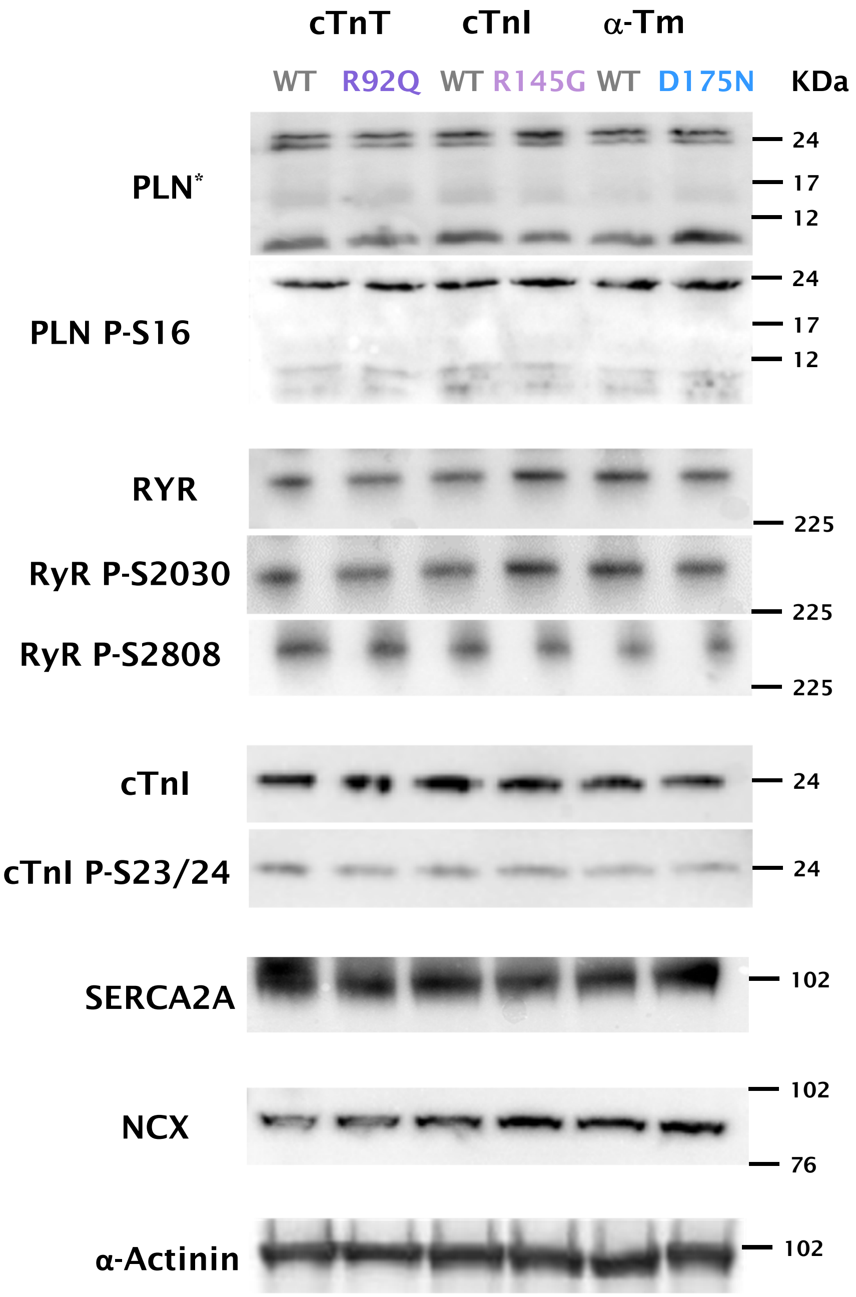

Figure S9. Absolute levels of Ca^{2+} handling and myofilament proteins are unchanged by HCM mutant protein expression and pacing. Western blotting of, the Ca^{2+} handling proteins PLN, RyR, TnI, NCX, RyR and SERCA2A, and the myofilament protein cTnI showed no change in band intensities of all WT and mutants infected cardiomyocyte preparations when normalised to α-Actinin loading controls. Control PLN image marked *, has been reused from Figure 5E. There was also no change to the relative levels of Phospho-Serine 16 PLN, Phospho-Serine 2030 and 2808 RyR and Phospho-Serine 23/24 cTnI.

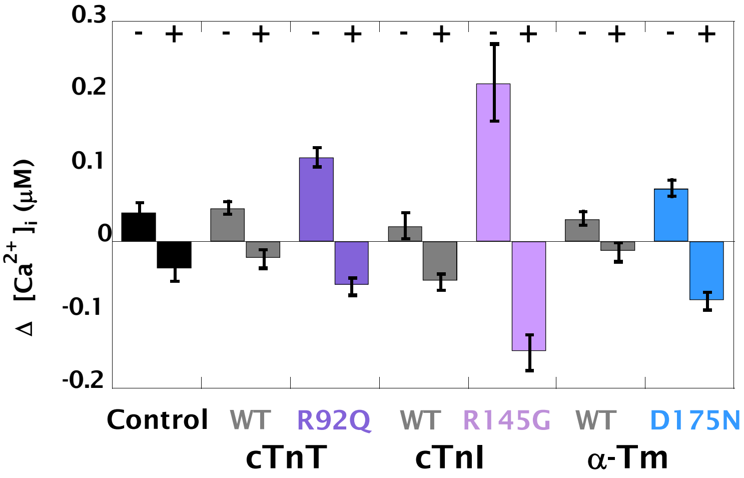

Figure S10. The breakdown of Δ[Ca^{2+}]$_i$ used to calculate RyR leak. Bar graph shows the Δ[Ca^{2+}]$_i$ in the absence of field stimulation, taken from RyR leak experiments in either 0Na0Ca solution (-) or 0Na0Ca solution containing 1μM tetracaine (+). The difference between the two Δ[Ca^{2+}]$_i$ for each cell was used to calculate the leak rate expressed in Figure 6B.

A

	n	Fractional Sarcomere Shortening (%)	Diastolic Sarcomere Length (μm)	Amplitude of Sarcomere Shortening (μm)	Time to 50% Peak Contraction (sec)	Time to 50% Relaxation (sec)	τ Relaxation (sec)
**T0hr**	*58*	3.095±0.294	1.906±0.009	0.059±0.009	0.061±0.005	0.089±0.010	0.072±0.008
T24hr	*62*	2.370±0.224	1.899±0.010	0.045±0.012	0.070±0.004	**0.149±0.011** ***	**0.114±0.012** ***
**T48hr**	*38*	2.522±0.135	1.903±0.011	0.048±0.007	**0.163±0.008** ***	**0.249±0.010** ***	**0.201±0.008** ***

B

	n	Calcium Transient Amplitude (μM)	Diastolic [Calcium] (μM)	Systolic [Calcium] (μM)	Time to 50% SR Release (sec)	Time to 50% SR Reuptake (sec)	τ Decay (Sec)
**T0hr**	*58*	0.736±0.012	0.245±0.012	0.981±0.016	0.021±0.002	0.148±0.005	0.113±0.004
T24hr	*62*	0.704±0.014	0.246±0.022	0.950±0.015	0.023±0.002	**0.194±0.009** ***	**0.152±0.009** ***
**T48hr**	*38*	0.743±0.018	0.234±0.007	0.977±0.012	**0.028±0.002** *	**0.247±0.009** ***	**0.199±0.008** ***

Table S1. Average extracted parameters from sarcomere shortening and Ca^{2+} transients of fura2 loaded cardiomyocytes during culture. Parameters with standard error and significance were taken from the sarcomere shortening curves in Figure S4A and Ca^{2+} transient curves in Figure S4B for tables A and B respectively. Significance values comparing freshly isolated cells with cells cultured for 24 and 48 hours are $p<0.001$=***, $p<0.01$=** and $p<0.05$=*.

		K_d (μM)	B_{max} (μM)	Relative Buffering Capacity
**Control**	41	1.629±0.312	161.8±36.57	-
WT cTnT	*20*	1.785±0.306	125.2±19.03	1.000±0.269
R92Q cTnT	*23*	**1.042±0.119** *	157.2±19.75	**1.753±0.247** **
WT cTnI	*9*	1.214±0.144	133.8±36.50	1.000±0.189
R145G cTnI	*11*	**0.547±0.179** *	120.8±25.84	**2.151±0.301** *
WT α-TM	*12*	1.888±0.485	226.3±54.18	1.000±0.237
D175N α-TM	*14*	**0.844±0.152** *	170.7±28.09	**2.254±0.365** *

Table S2. Average extracted parameters from Ca^{2+} buffering measurements. Parameters with standard error and significance were calculated from average Ca^{2+} buffering in Figure 2A using the equation Total $Ca^{2+} = \{B_{max} * [Ca^{2+}]_i / (K_d + [Ca^{2+}]_i)\} + B_{min}$. Significance values comparing wild type with HCM mutant values are $p<0.001$=***, $p<0.01$=** and $p<0.05$=*.

A

	n	Calcium Transient Amplitude (μmnol/L)	Diastolic [Calcium] (μM)	Systolic [Calcium] (μM)	Time to 50% peak Ca^{2+} transient (sec)	Time to 50% Ca^{2+} Decay Time (sec)	τ Decay (sec^{-1})
**Control**	*38*	0.796±0.014	0.234±0.007	0.951±0.008	0.045±0.005	0.247±0.009	0.233±0.002
WT cTnT	*41*	0.742±0.009	0.242±0.010	0.938±0.015	0.047±0.004	0.285±0.009	0.260±0.003
R92Q cTnT	*93*	0.703±0.013	**0.417±0.004** ***	**1.125±0.013******	0.051±0.003	**0.321±0.009** ***	**0.301±0.002******
WT cTnI	*85*	0.829±0.025	0.229±0.021	1.058±0.007	0.042±0.002	0.249±0.017	0.236±0.002
R145G cTnI	*101*	0.833±0.014	**0.536±0.026** ***	**1.309±0.015** ***	0.043±0.003	**0.297±0.014** ***	**0.262±0.003** ***
WT α-TM	*43*	**0.878±0.037** #	**0.189±0.012** #	0.969±0.070	0.056±0.007	0.259±0.018	0.253±0.005
D175N α-TM	*57*	**1.147±0.031** ***	**0.383±0.018** ***	**1.281±0.082** **	0.054±0.005	**0.273±0.014** *	**0.280±0.003******

B

	n	Fractional Sarcomere Shortening (%)	Diastolic Sarcomere Length (μm)	Amplitude of Sarcomere Shortening (μm)	Time to 50% Peak Contraction (sec)	Time to 50% Relaxation (sec)	τ Relaxation (sec^{-1})
**Control**	*48*	6.488±0.156	1.865±0.009	0.111±0.008	0.089±0.003	0.157±0.007	0.116±0.001
WT cTnT	*42*	6.465±0.214	1.862±0.006	0.110±0.006	0.084±0.003	0.156±0.004	0.111±0.001
R92Q cTnT	*87*	6.199±0.218	**1.832±0.007** ***	0.114±0.007	0.088±0.006	**0.184±0.004** ***	**0.135±0.001******
WT cTnI	*75*	6.432±0.107	1.870±0.007	0.112±0.004	0.087±0.004	0.159±0.006	0.120±0.003
R145G cTnI	*75*	**4.167±0.082** ***	**1.776±0.007** ***	**0.064±0.003** ***	**0.076±0.002** *	**0.196±0.004** ***	**0.140±0.001******
WT α-TM	*69*	**6.028±0.231** #	**1.898±0.006** ###	**0.969±0.004** ##	0.099±0.006	**0.175±0.006** ###	**0.120±0.003** ###
D175N α-TM	*58*	**6.414±0.262** **	**1.869±0.007** ***	**0.101±0.006** ***	0.082±0.004	**0.185±0.009** *	**0.129±0.002****

C

	n	Fractional Sarcomere Shortening (%)	Diastolic Sarcomere Length (μm)	Amplitude of Sarcomere Shortening (μm)	Time to 50% Peak Contraction (sec)	Time to 50% Relaxation (sec)	τ Relaxation (sec)
**Control**	*82*	2.601±0.075	1.978±0.008	0.048±0.003	0.163±0.008	0.249±0.010	0.256±0.005
WT cTnT	*41*	2.591±0.067	1.976±0.009	0.043±0.003	0.159±0.007	0.259±0.011	0.285±0.005
R92Q cTnT	*93*	**1.618±0.100** ***	**1.929±0.007** ***	**0.030±0.005** **	**0.127±0.007** *	**0.278±0.009** ***	**0.321±0.003****
WT cTnI	*85*	2.839±0.071	1.977±0.003	0.051±0.001	0.141±0.007	0.255±0.008	0.295±0.002
R145G cTnI	*101*	**1.457±0.061** ***	**1.868±0.005** ***	**0.027±0.001** ***	**0.118±0.004** ***	**0.282±0.013** ***	**0.356±0.003******
WT α-TM	*43*	2.711±0.180	**2.000±0.010** #	0.046±0.003	**0.191±0.013** ###	**0.289±0.021** ##	0.306±0.008
D175N α-TM	*57*	**3.659±0.259** ***	**1.927±0.008** ***	**0.060±0.004** ***	0.196±0.011	**0.352±0.014** *	**0.368±0.002***

Table S3. Average extracted parameters from Ca^{2+} transient and sarcomere length measurements. Parameters with standard error and significance in were taken from Ca^{2+} transients in figure 3A (A), sarcomere length measurements in figure 3D (B) and sarcomere length measurements from fura2 loaded cells in Figure S7 (C). Significance values comparing uninfected control with wild type values are p<0.001= ###, p<0.01= ## and p<0.05= #, whilst significance values comparing wild type with HCM mutant values are p<0.001=***, p<0.01=** and p<0.05=*.

	n	$I_{Ca2+,L}$ Peak (pA/pF)	$I_{Ca2+,L}$ Integral	$I_{Ca2+,L}$ t_{fast} Decay (ms)	$I_{Ca2+,L}$ t_{slow} Decay (ms)	I_{NCX} Peak (pA/pF)
**Control**	*33*	-4.997±0.987	8.247±0.968	8.923±1.002	125.2±15.98	-1.004±0.125
**WT cTnT**	*22*	-5.865±0.785	9.655±1.394	8.654±0.911	97.51±8.390	-1.220±0.170
**R92Q cTnT**	*25*	-3.940±0.521	9.148±0.984	10.86±1.004	**122.1±10.10** *	**-1.861±0.227** *
**WT cTnI**	*11*	-7.201±1.375	8.654±1.080	7.265±1.666	**49.67±10.93** #	-0.952±0.117
**R145G cTnI**	*12*	-5.044±1.404	12.69±3.900	7.220±0.871	96.71±22.98	**-1.792±0.317** *
**WT α-TM**	*18*	-3.581±0.544	6.328±0.903	9.239±0.810	**290.9±80.58** #	**-0.655±0.080** #
**D175N α-TM**	*23*	-2.577±0.546	5.327±0.950	11.43±0.952	260.0±46.51	**-1.007±0.107** *

Table S4. Average extracted parameters from L-type Ca^{2+} and NCX current measurements acquired during Ca^{2+} buffering experiments. L-Type Ca^{2+} current parameters taken from Figure S8 NCX peak current taken from Figure 3. Significance values comparing wild type with HCM mutant values are p<0.001=***, p<0.01=** and p<0.05=*. Significance values comparing uninfected control with wild type infected values are p<0.001=###, p<0.01=## and p<0.05=#.

A

	n	$[Ca^{2+}]_i$ SR Load (μM)	$[Ca^{2+}]_i$ Fractional Release (%)	$[Ca^{2+}]_i$ Caffeine Tau Decay (sec)	$[Ca^{2+}]_i$ Trasient Amplitude (μM)	$[Ca^{2+}]_i$ Tau Decay (Sec)	$[Ca^{2+}]_i$ SERCA2 Activity μM/sec
**Control**	*56*	2.100±0.090	37.87±1.571	1.141±0.079	0.772±0.015	0.235±0.008	3.076±0.269
WT cTnT	*20*	2.103±0.145	39.23±2.588	1.100±0.064	0.754±0.019	0.2599±0.013	2.938±0.199
R92Q cTnT	*23*	**1.623±0.198** *	**62.97±10.88** *	**1.420±0.142** *	0.708±0.027	**0.301±0.012** *	3.521±0.402
WT cTnI	*9*	2.111±0.157	38.73±2.838	1.114±0.072	0.792±0.035	0.246±0.032	3.167±0.409
R145G cTnI	*11*	**1.557±0.176** *	**55.16±5.387** *	**1.482±0.130** *	0.778±0.031	**0.341±0.023** *	3.001±0.279
WT α-TM	*12*	**2.201±0.128**	36.73±3.735	1.038±0.092	0.753±0.038	0.259±0.015	3.328±0.485
D175N α-TM	*14*	**1.636±0.177** *	**63.09±7.572** **	1.207±0.080	**0.899±0.041** *	**0.293±0.017** *	2.743±0.263

B

	n	$[Ca^{2+}]_{total}$ SR Load (μM)	$[Ca^{2+}]_{total}$ Fractional Release (%)	$[Ca^{2+}]_{total}$ Caffeine Tau Decay (sec)	$[Ca^{2+}]_{total}$ Trasient Amplitude (μM)	$[Ca^{2+}]_{total}$ Tau Decay (Sec)	$[Ca^{2+}]_{total}$ SERCA2 Activity (μM/sec)
**Control**	*56*		61.36±8.998	1.057±0.045	46.25±7.254	0.224±0.013	3.352±0.187
WT cTnT	*20*	64.24±8.874	47.50±3.200	1.057±0.078	30.34±5.023	0.234±0.018	3.056±0.189
R92Q cTnT	*23*	**98.84±12.08** *	56.98±3.829	**0.758±0.082** *	**66.53±10.01** *	**0.178±0.021** *	**4.181±0.324** *
WT cTnI	*9*	75.84±11.34	73.65±9.587	0.991±0.087	51.32±7.973	0.219±0.024	3.360±0.309
R145G cTnI	*11*	**117.1±11.39** *	76.81±7.880	**0.724±0.077** *	**85.86±9.883** *	**0.162±0.029** *	**4.432±0.274** *
WT α-TM	*12*	83.26±7.513	70.65±12.28	0.958±0.085	53.64±6.078	0.209±0.017	3.647±0.214
D175N α-TM	*14*	**134.6±12.14** **	61.92±6.393	**0.706±0.075** *	**80.45±8.721** *	**0.154±0.021** **	**5.200±0.236** *

Table S5. Average extracted parameters from $[Ca^{2+}]_i$ and $[Ca^{2+}]_{total}$ SR Load and Ca^{2+} transients following 5 second pause. Parameters with standard error and significance in A were taken from SR load measurements and the preceding Ca^{2+} transients using $[Ca^{2+}]$i (free calcium) observations during caffeine spritz shown in Figure 5A insets following a 5 second pause in pacing. Parameters with standard error and significance in B were taken from SR load measurements and the preceding Ca^{2+} transients using $[Ca^{2+}]_{total}$ calculations during caffeine applications shown in Figure 5A. SR load was estimated using a direct spritz of 10 mM caffeine following a 5 second pause from electrical stimulation at 0.5 hz. Significance values comparing wild type with HCM mutant values are $p<0.001$=***, $p<0.01$=** and $p<0.05$=*.

	n	Caffeine Transient Amplitude (μmol/L)	Fractional SR Release (%)	τ Decay (sec)	SERCA2 Activity (sec^{-1})
**Control**	*56*	2.616±0.141	37.02±1.851	1.141±0.079	3.076±0.269
WT cTnT	*45*	2.675±0.136	36.55±2.043	1.100±0.064	2.938±0.199
**R92Q cTnT**	*39*	**2.328±0.106** **	**50.81±2.891** ***	**1.420±0.142** *	3.521±0.402
WT cTnI	*38*	2.655±0.163	32.23±2.171	1.114±0.072	3.167±0.409
R145G cTnI	*30*	**1.819±0.116** ***	**58.65±3.302** ***	**1.482±0.130** *	3.001±0.279
WT α-TM	*26*	**3.059±0.264** *	**31.08±2.202***	1.038±0.062	3.328±0.485
D175N α-TM	*36*	**2.344±0.121** ***	**46.46±2.613** ***	**1.207±0.050** *	2.743±0.263

Table S6. Average extracted parameters from $[Ca^{2+}]_i$ SR Load measurements acquired without pause. Parameters with standard error and significance were taken from caffeine transients acquired independently of experiments detailed in Figure 5 and Table S5. Caffeine was applied immediately following deactivation of the pacing train. Significance values comparing wild type with HCM mutant values are p<0.001=***, p<0.01=** and p<0.05=*.

	n	$\Delta_i[Ca^{2+}]$ - tet (μM)	$\Delta_i[Ca^{2+}]$ + tet (μM)	Caffeine Amplitude - tet (μM)	Caffeine Amplitude + tet (μM)	RyR Leak (nM / Sec)	Leak / Load
**Control**	*30*	0.039± 0.012	-0.037±0.016	2.201±0.137	2.038±0.186	0.755±0.246	$0.202x10^{-3}±0.035x10^{-3}$
WT cTnT	*30*	0.046±0.008	-0.024±0.012	1.987±0.215	2.014±0.201	0.759±0.155	$0.229x10^{-3}±0.041x10^{-3}$
**R92Q cTnT**	*30*	**0.115±0.013*****	**-0.062±0.011****	**2.364±0.110*****	**2.726±0.144*** / #**	**3.542±0.272 *****	**$0.454x10^{-3}±0.105x10^{-3}$ *****
WT cTnI	*30*	0.021±0.018	-0.055±0.011	2.221±0.136	2.284±0.236	0.833±0.186	$0.096x10^{-3}±0.040x10^{-3}$
R145G cTnI	*30*	**0.217±0.053*****	**-0.152±0.024*****	**3.000±0.190*****	**3.293±0.202*** / #**	**5.201±0.472 *****	**$0.689x10^{-3}±0.027x10^{-3}$ ****
WT α-TM	*30*	0.031±0.010	-0.014±0.013	1.956±0.099	2.072±0.158	0.518±0.086	$0.155x10^{-3}±0.076x10^{-3}$
**D175N α-TM**	*30*	**0.072±0.011*****	**-0.081±0.012*****	**3.026±0.141*****	**3.938±0.0.184*** / ###**	**3.077±0.322 *****	**$0.208x10^{-3}±0.068x10^{-3}$ ***

Table S7. Average extracted parameters from RyR leak experiments. Parameters with standard error and significance were taken from RyR leak experiments transients in Figure 6. Significance values comparing wild type with HCM mutant values are p<0.001=***, p<0.01=** and p<0.05=*. Significance values comparing treatment with 1 μM tetracaine with untreated values are p<0.001= ###, p<0.01= ## and p<0.05= #.

	WT cTnT	R92Q cTnT	WT cTnI	R145G cTnI	WT α-TM	D175N α-TM
**NFAT P-Ser165 Unpaced**	1	1.014±0.098	1	1.236±0.214	1	0.925±0.099
**NFAT P-Ser165 Paced**	1	**0.317±0.148** ***	1	**0.270±0.153** ***	1	**0.151±0.116** ***
**ΔNFAT P-Ser165 Paced vs Unpaced (%)**	2.587±5.695	**-70.14±2.987** ***	-5.987±4.874	**-82.48±6.692** ***	6.247±3.692	**-98.78±4.587** ***
**ERK P-Thr-202/Tyr204 Unpaced**	1	**1.552±0.168** *	1	**1.659±0.145** **	1	**1.666±0.177** *
**ERK P-Thr-202/Tyr204 Paced**	1	**1.604±0.221** *	1	**1.454±0.066** **	1	**1.302±0.079** *
**ΔERK P-Thr-202/Tyr204 Paced vs Unpaced (%)**	1340±270.3	**1936±198.4** *	1546±370.1	**2357±264.7** *	1546±294.9	**2414±380.5** *
**Nuclear NFAT IF Unpaced**	1.000±0.141	**2.627±0.154** **	1.000±0.163	0.874±0.180	1.000±0.197	1.427±0.224
**Nuclear NFAT IF Paced**	1.000±0.210	**9.593±0.208** ***	1.000±0.185	**3.341±0.189** ***	1.000±0.199	**2.358±0.183** ***
**ΔNuclear NFAT IF Paced vs Unpaced (%)**	-8.712±0.405	**114.4±14.49** ***	10.11±8.302	**133.6±20.25** ***	27.86±9.195	**95.61±11.63** ***
**Nuclear ERK IF Unpaced**	1.000±0.243	**0.154±0.229** *	1.000±0.197	0.554±0.127	1.000±0.645	**-1.663±0.595** *
**Nuclear ERK IF Paced**	1.000±0.089	1.158±0.096	1.000±0.262	**1.572±0.138** *	1.000±0.646	**7.225±0.599** ***
**ΔNuclear ERK IF Paced vs Unpaced (%)**	31.38±4.196	**52.03±4.507** **	13.06±11.66	**52.48±6.133** **	19.59±5.651	**76.80±7.375** ***

Table S8. Densitometry and image analysis comparing NFAT and ERK phosphorylation levels and NFAT and ERK nuclear localisation in paced vs unpaced cardiomyocytes comparing WT and mutant infected cardiomyocytes. Relative changes between wild type and mutant infected cells for NFAT and ERK phosphorylation at serine-165 and Threonine-202 / Tyrosine-204, for unpaced (rows 1 and 4) and paced (0.5 hz for 8 hours) (rows 2 and 5) cardiomyocytes respectively. Row 3 and 6 gives the % change in phosphorylation when comparing paced (0.5 hz for 8 hours) vs unpaced preparations for NFAT and ERK phosphorylation respectively. Below is given the relative changes between wild type and mutant infected cells for NFAT and ERK nuclear localisation compared to cytosolic background, for unpaced (rows 7 and 10) and paced (0.5 hz for 8 hours) (rows 8 and 11) cardiomyocytes respectively. Rows 9 and 12 gives the % change in nuclear NFAT and ERK upon pacing (0.5 hz for 8 hours) respectively Significance values comparing wild type with HCM mutant values are p<0.001=***, p<0.01=** and p<0.05=*.
